# Circulating inflammatory cytokines and psoriasis risk: A systematic review and meta-analysis

**DOI:** 10.1371/journal.pone.0293327

**Published:** 2023-10-26

**Authors:** Xiao-Qing Liu, Pei-Lin Zhou, Xin-Yu Yin, Ai-Xue Wang, Da-Hu Wang, Yun Yang, Qiang Liu

**Affiliations:** Department of Dermatology, The Second Hospital of Hebei Medical University, Shijiazhuang, Hebei, China; Lerner Research Institute - Cleveland Clinic, UNITED STATES

## Abstract

**Background:**

Psoriasis is a systemic immune-mediated chronic inflammatory skin disease; its systemic manifestations and periodic recurrence negatively affect a patient’s quality of life. Inflammatory cytokines are known to have an important role in the onset and progression of psoriasis, however, data on the association between circulating inflammatory cytokines and psoriasis risk is inconclusive. Here, we explore the relevance of circulating proinflammatory factors to the pathogenesis of psoriasis using a meta-analysis.

**Objective:**

To explore the association between circulating levels of inflammatory factors and psoriasis to elucidate the mechanisms underlying psoriasis and improve clinical diagnosis and treatment.

**Methods:**

We systematically retrieved articles published in PubMed, EMBASE, the Cochrane Library and the Web of Science from the establishment of each database to January 2023. The standard mean difference (SMD) in cytokine levels of individuals with psoriasis and healthy controls was used to check for correlations between circulating inflammatory factor levels and psoriasis.

**Results:**

Fifty-seven studies, with data from 2838 patients, were retrieved and included in the meta-analysis. Eleven inflammatory factors were studied (circulating interleukin-2 (IL-2), IL-4, IL-12, IL-17, IL-18, IL-22, IL-23, IL-35, IL-36, transforming growth factor-beta (TGF-β) and gamma-interferon (IFN-γ)). Of these, IL-2 [SMD = 1.29 (95% CI: 0.61–1.97; P <0.001)], IL-17 [SMD = 0.71 (95% CI: 0.12–1.30; P = 0.018)], IL-18 [SMD = 1.27 (95% CI: 0.64–1.90; P <0.001)], and IFN-γ [SMD = 1.90 (95% CI: 1.27–2.52; P <0.001)] levels had significant correlations with psoriasis.

**Conclusion:**

Increased serum concentrations of the circulating inflammatory cytokines IL-2, IL-17, IL-18 and IFN-γ were significantly correlated with psoriasis.

## Introduction

Psoriasis is a skin disorder characterized by an inflammatory process mediated by immune cells. Its typical clinical manifestation includes inflammatory erythema, scales and many complications, including metabolic syndrome and cardiovascular disease [1–3]. The disease often presents with systemic or widespread symptoms that are difficult to treat and adversely affect the patient’s quality of life. At present, psoriasis is thought to be caused by various types of cells (including neutrophils, dendritic cells, macrophages, T lymphocytes, mast cells and keratinocytes) and their production of circulating inflammatory factors [4–6]. The resulting circulating inflammatory factors are thought to play a central part in the pathogenesis of psoriasis, and the changes of inflammatory factors in serum are tightly associated with the occurrence of psoriatic lesions and the presence of further aggravation [7–9]. Therefore, as important mediators of psoriasis, inflammatory factors have attracted increased attention among researchers actively searching for possible treatments and diagnostic biomarkers [7, 10–12].

Previous experimental studies indicate that some circulating inflammatory factors are elevated in patients suffering from psoriasis compared to healthy controls and that these circulating inflammatory factors may be associated with the risk of psoriasis [13, 14]. However, the small sample sizes of previous studies limit confidence in this association. We thus performed a meta-analysis to gain insight into the link between circulating inflammatory factors and psoriasis risk to explore their value for treating psoriasis in clinical practice. In this study, potentially relevant circulating inflammatory factors were systematically and comprehensively tested, including serum inflammatory markers interleukin (IL)-2, IL-4, IL-18, IL-12, IL-17, IL-22, IL-23, IL-35, IL-36, gamma-interferon (IFN-γ) and transforming growth factor-beta (TGF-β) (Table 1). We used the anti-inflammatory factors IL-35 and TGF-β as controls for the generalized elevation of all factors. Our goal was to assess whether levels of systemic markers of inflammation differ in individuals with psoriasis compared to those in healthy subjects and to measure the extent of such changes, thereby providing a clinical reference for the search and clarification of new inflammatory factors.

**Table 1:** Role of selected circulating inflammatory factors in psoriasis.

factors	detail
**IL-2**	The pro-inflammatory factor secreted by Th1 cells is a factor on which the growth and survival of T cells depend, and it can enhance the proliferation of T lymphocytes and the killing activity of NK cells [15, 16].
**IL-4**	It is a multifunctional and pleiotropic pro-inflammatory factor, mainly produced by activated Th2 cells, which can promote the differentiation and maturation of dendritic cells (DC) [17].
**IL-12**	An important pro-inflammatory factor that regulates the imbalance of Th1/Th2 ratio, can promote the differentiation of Th0 cells into Th1 cells, and induce Th1 cells to secrete IL-2 and IFN-γ [18, 19].
**IL-17**	The marker factor of Th17 cells is a pro-inflammatory factor, which can induce the activation of T cells and macrophages to promote inflammation. IL-17 has a synergistic effect with IL-22, and IL-17 can induce the autocrine of IL-22 [20, 21].
**IL-18**	The pro-inflammatory factors mainly secreted by macrophage cells activate Th1 lymphocytes and macrophages, and promote the production of IL-2, TNF-α, IFN-γ, etc. [22] to inhibit the secretion of IL-4 and IL-10. IL-18 and IL-12 have a synergistic effect and jointly induce, promote and regulate the differentiation and maturation of Th1 cells [23].
**IL-22**	An important immune pro-inflammatory factor mainly produced by activated Th17 cells and Th22 cells [24]. It can also be produced by Th1 cells, etc., to promote inflammation of psoriasis and thickening of the epidermis [25]. IL-22 and IL-17 synergistically promote keratinization The cells express IL-8 and CCL-20, which induce the infiltration of inflammatory cells [26].
**IL-23**	The pro-inflammatory factors secreted by Th17 cells enhance and maintain the activity of Th17 cells in the later stage [24]. IL-23 promotes the excessive proliferation and differentiation of keratinocytes, and promotes the secretion of IL-17 and IL-6 [27].
**IL-35**	The only inhibitory factor specifically produced by Treg cells [28]. Expressed rapidly in the late stage of inflammation, it is necessary for Treg cells to exert their inhibitory function, and can promote the proliferation of Treg cells and inhibit the differentiation of Th17 cells and the secretion of related factors. And can inhibit the expression of pro-inflammatory factors, significantly up-regulate the expression of IL-10 [29].
**IL-36**	It is a pro-inflammatory factor that can activate CD4+ T lymphocytes, dendritic cells, and keratinocytes, and induce T cell proliferation to produce circulating inflammatory factors [27, 29]. In the skin, keratinocytes are the main effector cells [30].
**IFN-γ**	The main pro-inflammatory factor produced by Th1 cells can activate dendritic cells to produce IL-23 and stimulate Th17 cell responses to produce inflammatory factors [25].
**TGF-β**	It is an inhibitory factor, mainly produced by Treg cells, which affects the differentiation of early Th17 cells and can weaken the proliferation of keratinocytes [31].

## Materials and methods

### Study registration

This research was performed according to the Preferred Reporting Items for Systematic Reviews and Meta-Analyses (PRISMA) guidelines [32] and is registered in the PROSPERO database (CRD42023395263).

### Search strategy

We conducted a systematic review of articles released since the creation of the PubMed, EMBASE, Cochrane Library and Web of Science databases up until January 2023. No restrictions were placed on language or source during the initial search of published articles. Medical Subject Headings (MeSH) together with free terms were as keywords for literature searching. the example of the PubMed search strategy, the main search terms were: "Interleukin-2" OR "Interleukin-4", "Interleukin-12" OR "Interleukin-17" OR "Interleukin-18" OR "Interleukin-22" OR "Interleukin-23" OR "Interleukin-35" OR "Interleukin-36", "Transforming Growth Factor β (TGF-β)" OR "interferon-gamma (IFN-γ)" AND "case-control work" OR "cross-sectional work" OR "cohort work" AND "psoriasis. The supplementary material provides comprehensive details of the search strategy employed. Independently manual screening and data collection by multiple researchers to ensure transparency and reproducibility of methods. This rigorous process also serves to validate the qualification of the selected documents.

### Selection criteria

Literature sources for the meta-analysis had to meet the following criteria: (a) the case group was patients with clinically confirmed adult psoriasis (>18 years old) who had not received any systematic therapies for a minimum of half a month or patients with other systemic and immune diseases, The inclusion criteria did not impose any restrictions based on gender, disease severity, or medical history; the control group was healthy people without psoriasis, other systemic diseases or other skin diseases; (b) clinical case-control, cross-sectional studies or clinical cohort studies; (c) the outcome/measurement indicators were concentration or plasma level of factors in the peripheral serum of the research subjects, and data were given as mean ± standard deviation or could be converted to mean ± standard deviation; (d) where duplicate data for reports by the same author were found, the most complete and newly published research data were used.

Exclusion criteria were: (a) research measuring circulating inflammatory factors at post-treatment levels or measuring cytokines in tissues; (b) reviews, animal studies, clinical guidelines, letters to the editor, or cases report; (c) duplicate publications with similar data; (d) studies where original research data were incomplete or outcome data could not be extracted.

### Data extraction

The following characteristics of each qualifying research study were noted: name of the first author, year of publication, country, number of patients and control group subjects, sex ratio, age, sample type, Psoriasis type, PASI(psoriasis area and severity index),inflammatory factor detection method and whether the inflammatory indicators are connected to psoriasis.

### Quality assessment

Studies enrolled were assessed for their quality using the NIH (National Institutes of Health) [33] observational cohort and a cross-sectional research quality evaluation instrument to evaluate the quality of eligible studies. The scale was designed specifically for different studies; it primarily focuses on the internal validity of research concepts to test for flaws in research methods and implementation protocols. In all cases, quality assessments was done independently by two reviewers for each publication. Disagreements, if present, were discussed and resolved by a third investigator. The NIH total score ranges from 0 to 14 and is classified as poor (0–5), fair (6–10), or good (11–14).

### Statistical analysis

The standard mean difference (SMD) in levels of circulating inflammatory factors between psoriasis patients and healthy subjects and its 95% CI were employed to describe the association between factors and psoriasis risk. The heterogeneity between studies was estimated by the Cochrane Q test and the *I*^*2*^ statistic (*I*^*2*^ = 0–25%, data are homogeneous; *I*^*2*^ = 25–50%, mild heterogeneity; *I*^*2*^ = 50–75%, moderate heterogeneity; *I*^*2*^ = 75–100%, high heterogeneity). We found several separate reports had medium or high levels of heterogeneity (*I*^*2*^ ≥50% or *P* ≤0.10), and thus a random-effects model was employed. When heterogeneity was large, a subgroup analysis was performed to explore underlying causes of heterogeneity and a sensitivity analysis was used to assess the usability and reliability of the results. Publishing bias was examined using Egger’s test and funnel plots. If the two-sided *P*-value was <0.05, the effect of the circulating proinflammatory mediators on the risk of psoriasis was considered statistically significant. The quantitative meta-analysis was performed using STATA 15.1.

## Results

### Characterization and quality assessment of incorporated research

**Fig 1:**
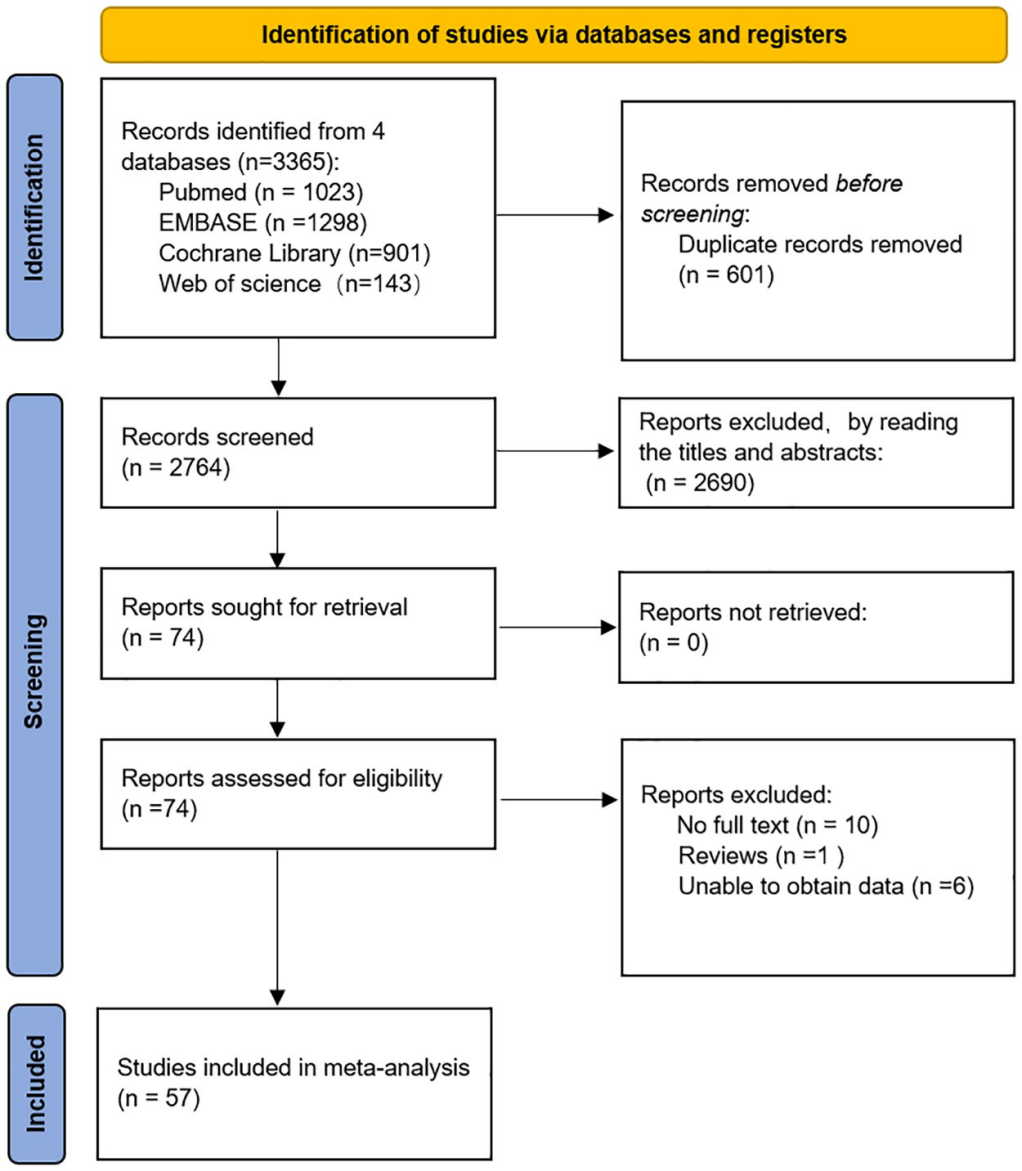
Preferred Report Items in Systematic Reviews and Meta-Analyses (PRISMA).

The literature was searched in detail (Fig 1). During the initial searches, 3365 articles were identified according to the criteria for selection. After reviewing the titles and abstracts, 601 duplicate articles, 84 reviews, systematic reviews, animal experiments, and 2606 non-relevant papers were eliminated. Following the review of the whole text, we eliminated one review, 10 articles where the full text was not found and six articles where data were not available or were insufficient. Finally, 57 studies were included in the meta-analysis, including 2838 cases and 2283 controls. Most of the projects measured the concentration of infectious cytokines with the use of ELISA, although a few projects utilized alternative approaches. In terms of qualitative scoring, according to the NIH quality score, all 57 studies rated 7 (Table 2).

**Table 2:** Global features of 57 papers on the relationship between circulating inflammatory cytokines and psoriasis risks.

	First author	Year	Country/city	Cases			Controls			Age, years(mean±SD)		Psoriasis type	PASI (mean±SD)	Sample type	Measurement method	Study type	Quality score
				nP	M	F	nC	M	F	Cases	Controls						
**IL-2**	Mannangi,N.B. [34]	2022	India	110	_	_	110	_	_	_	39.4±11.1	3	Mild: 38.8±8.5 Moderate: 39.9±12.7 Severe: 41.4±11.1	serum	ELISA	1	6
	Khandpur,S. [35]	2018	India	30	18	12	15	10	5	29.67±10.22	29.42±9.88	3	5.78±4.56	serum	ELISA	2	11
	Takahashi,H. [36]	2010	Japan	122	81	41	78	54	24	47.5 ± 7.6	38.6	3	7.3 ± 4.2	serum	ELISA	1	5
	Kaur,S. [37]	2012	Tartu	58	35	23	58	28	30	41.7 ± 12.0	41.4 ± 12.1	2	9.5±5.6	serum	High-sensitivity array	1	8
	el Barnawi, N.Y. [38]	2001	Benghazi	25	10	15	25	11	14	33± 12	31±11	1	_	serum	ELISA	2	11
	Choe,Y.B. [39]	2012	Seoul, Korea	71	41	30	15	_	_	36.3±14.0	34.2±12.6	3	7.9±5.2	serum	ELISA	1	8
**IL-4**	Chen,J. [40]	2014	Shanghai	15	8	7	16	8	8	46.13±16.24	42.31±13.61	1	7.4 ±3.3	serum	ELISA	1	11
	Khandpur,S. [35]	2018	India	30	18	12	15	10	5	29.67±10.22	29.42±9.88	3	5.78±4.56	serum	ELISA	2	11
	el Barnawi,N. Y. [38]	2001	Benghazi	25	10	15	25	11	14	33± 12	31±11	1	_	serum	ELISA	2	11
	Verghese,B. [41]	2011	India	30	17	13	30	_	_	34.5 ± 13.3	_	3	_	serum	ELISA	1	8
**IL-12**	Takahashi,H. [36]	2010	Japanese	122	81	41	78	54	24	47.5 ± 7.6	38.6	3	7.3 ± 4.2	serum	ELISA	1	5
	Michalak-Stoma,A. [42]	2013	Poland	60	50	10	30	_	_	45.6±13.2	_	3	15.7 ± 9.7	serum	ELISA	1	8
	Kyriakou,A. [43]	2014	Greece	32	9	23	32	_	_	44.53±15.60	_	2	_	Serum	ELISA	2	8
	Arican,O. [44]	2005	Turkey	30	18	12	23	_	_	35 ± 15.5	_	3	9.3 ± 8.15	Serum	ELISA	2	11
	Brito-Luna,M. J. [45]	2016	Mexico	24	11	13	14	6	8	44 ± 18.06	39 ± 9.44	2	_	Serum	ELISA	1	11
**IL-17**	Elbana,A.M. [46]	2022	Egypt	40	22	18	40	17	23	45.48±14.35	40.85±13.64	3	19.4 ± 8.11	Serum	ELISA	1	7
	Xuan,M.L. [47]	2015	China	62	_	_	20	_	_	_	37.25±12.78	3	12.84 ± 7.60	Serum	ELISA	1	7
	Takahashi,H. [36]	2010	Japan	122	81	41	78	54	24	47.5 ± 7.6	38.6	3	7.3 ± 4.2	Serum	ELISA	1	5
	Michalak-Stoma, A. [42]	2013	Poland	60	50	10	30	_	_	45.6±13.2	_	3	15.7 ± 9.7	Serum	ELISA	1	8
	Kyriakou,A. [43]	2014	Greece	32	9	23	32	_	_	44.53±15.60	_	2	_	Serum	ELISA	2	8
	Fotiadou,C. [48]	2015	Greece	35	28	7	20	_	_	47.0 ± 16.0	48.55±14.24	2	16.1 ± 7.2	Serum	flow cytometry	2	7
	Choe,Y.B. [39]	2012	Korea	71	41	30	15	_	_	36.3± 14.0	34.2±12.6	2	7.9 ± 5.2	Serum	ELISA	1	8
	Chhabra,S. [49]	2016	India	34	27	7	24	16	8	37.5± 13.5	27.4± 5.5	2	4.87±3	Serum	ELISA	1	7
	Akşan,B. [50]	2022	Germany	188	66	122	376	132	244	44.08±14.17	47.61 ± 12.0	3	_	Serum	ELISA	2	11
	Arican,O. [44]	2005	Turkey	30	18	12	23	_	_	35 ± 15.5	_	2	9.3 ± 8.15	Serum	ELISA	1	10
	Nassar,A.A. [51]	2022	Egypt	40	28	12	40	25	15	42.3 ± 13.7	40.8 ± 11.9	2	15.7± 10.4	Serum	ELISA	1	7
**IL-18**	Takahashi,H. [36]	2010	Japan	122	81	41	78	54	24	47.5 ± 7.6	38.6	3	7.3 ± 4.2	serum	ELISA	1	5
	Pietrzak,D. [52]	2018	Poland	85	85	0	65	65	0	47 ± 14	44 ± 13	3	17 ± 9	serum	ELISA	2	11
	Pietrzak,A. [53]	2003	Poland	12	6	6	10	5	5	29.9± 11.2	_	3	_	serum	ELISA	2	8
	Gangemi,S. [54]	2003	Italy	16	10	6	16	9	7	41.75±16.59	39.88± 15.53	3	35.81±18.80	serum	ELISA	2	6
	Arican,O. [44]	2005	Turkey	30	18	12	23	_	_	35 ± 15.5	_	2	9.3 ± 8.15	serum	ELISA	1	10
**IL-22**	Michalak-Stoma, A. [42]	2013	Poland	60	50	10	30	_	_	45.6±13.2	_	3	15.7 ± 9.7	Serum	ELISA	1	8
	Fotiadou,C. [48]	2015	Greece	35	28	7	20	_	_	47.0 ± 16.0	48.55±14.24	2	16.1 ± 7.2	Serum	flow cytometry	2	7
	Sobhan,M. R. [55]	2016	Iran	28	21	7	28	_	_	46.7±17.4	44.5±16.8	3	10.02±7.8	Serum	ELISA	2	7
	Hofny,E.R.M. [56]	2017	Egypt	25	_	_	25	_	_	_	_	1	_	Serum	ELISA	1	9
	Brito-Luna,M. J. [45]	2016	Mexico	24	11	13	14	6	8	44 ± 18.06	39 ± 9.44	2	_	Serum	ELISA	1	11
**IL-23**	Michalak-Stoma, A. [42]	2013	Poland	60	50	10	30	_	_	45.6±13.2	_	3	15.7 ± 9.7	Serum	ELISA	1	8
	Kyriakou,A. [57]	2014	Greece	32	9	23	32	_	_	44.53±15.60	_	2	_	Serum	ELISA	2	8
	Fotiadou,C. [48]	2015	Greece	35	28	7	20	_	_	47.0 ± 16.0	48.55±14.24	2	16.1 ± 7.2	Serum	flow cytometry	2	7
	Filiz,B. [58]	2019	Turkey	67	37	30	67	31	36	40.55±14.83	36.00±13.07	3	10.49±1.34	Serum	ELISA	1	11
	Chhabra,S. [49]	2016	India	34	27	7	24	16	8	37.5± 13.5	27.4± 5.5	2	4.87±3	Serum	ELISA	1	7
	Brito-Luna,M. J. [45]	2016	Mexico	24	11	13	14	6	8	44 ±18.06	39 ± 9.44	2	_	Serum	ELISA	1	11
**IL-35**	Elbana,A.M. [46]	2022	Egypt	40	22	18	40	17	23	45.48±14.35	40.85±13.64	3	19.4 ± 8.11	Serum	ELISA	1	7
	Chen,J. [59]	2021	China	30	17	13	30	18	12	33.24±11.83	34.35±13.05	1	10. 48 ± 3. 37	Serum	ELISA	1	11
**IL-36**	Sehat,M. [60]	2022	Iran	47	_	_	47	_	_	33.83±11.93	30.17±4.82	1	_	Serum	ELISA	1	7
	Chen,J. [59]	2021	China	30	17	13	30	18	12	33.24±11.83	34.35±13.08	1	10.48±3.37	Serum	ELISA	1	11
**IFN-γ**	Khandpur,S. [35]	2018	India	30	18	12	15	10	5	29.67±10.22	29.42±9.88	3	5.78±4.56	Serum	ELISA	2	11
	Mannangi,N. B. [34]	2022	India	110	_	_	110	_	_	_	39.4±11.1	3	_	Serum	ELISA	1	6
	Elbana,A.M. [46]	2022	Egypt	40	22	18	40	17	23	45.48±14.35	40.85±13.64	3	19.4 ± 8.11	Serum	ELISA	1	7
	Arican,O. [44]	2005	Turkey	30	18	12	23	_	_	35 ± 15.5	_	2	9.3 ± 8.15	Serum	ELISA	1	10
	Abdallah,M. A. [61]	2009	Egypt	21	10	11	15	_	_	_	_	3	25.8±15.1	Serum	ELISA	2	7
	Mawla, M. Y. M. A. [62]	2022	_	28	15	13	28	15	13	40.11±15.92	23.29±7.61	3	11.64±8.07	Serum	ELISA	1	7
	el Barnawi,N. Y. [38]	2001	Benghazi	25	10	15	25	11	14	33± 12	31±11	1	_	Serum	ELISA	2	11
**TGF-β**	Ahmed, B. T. [63]	2020	Iraq	100	52	48	50	19	31	42.4±14.8	46.3(±19.5)	1	_	Serum	ELISA	1	10
	Elbana,A.M. [46]	2022	Egypt	40	22	18	40	17	23	45.48±14.35	40.85±13.64	3	19.4 ± 8.11	Serum	ELISA	1	7
	Meki, A. R. [64]	2014	Arabia	58	36	22	22	11	11	30.17±1.406	29.36±1.88	1	_	Serum	ELISA	2	7
	Zaher, H. [65]	2009	Egypt	22	13	9	10	3	7	47.2 ± 15.8	40.7 ± 9.5	1	12.7 ± 8.4	Serum	ELISA	1	7

Cases nP, number of patients groups; Cases M, number of males in patients groups; Cases F, number of females in patients groups; Controls nC, number of controls; Controls M, number of males in control groups; Controls F, number of females in control groups; Age, years (mean ± SD). Cases, mean ± SD of the number in the case group; age, years (mean ± SD). type 1 psoriasis, common psoriasis; type 2 psoriasis, plaque psoriasis; type 3 psoriasis, others psoriasis forms (mixed or unspecified forms); PASI, psoriasis area and severity index; ELISA, enzyme-linked immunosorbent assay; Study type 1, The case control study; Study type 2, The cross-sectional study; Study type 3, The cohort studies. ’-’ indicates missing data.

### Circulatory inflammation factors and risks of psoriasis

#### Forest plot of the correlation between circulating inflammatory factors and the risk of psoriasis

Taking the correlation between IL-2 and the risk of psoriasis as an example, the meta-analysis uses SMD as the effect size; the Q test for heterogeneity (*I*^*2*^ = 93.1% >50%, *P* <0.001) indicates high heterogeneity among studies and thus a random effect analysis was performed. The pooled SMD from the meta-analysis was 1.29 (95% CI: 0.61–1.97; *P* < 0.001), showing that the levels of IL-2 in external serum were significantly greater in patients with psoriasis compared to healthy controls (Fig S1 in S1 File). Likewise, for the index of IL-17, the pooled efficacy of large and small SMD was 0.68 (95% CI: 0.14–1.23; *P* = 0.018), indicating that circulating IL-17 levels were slightly higher in individuals with psoriasis than in controls (Fig S2 in S1 File). Serum levels of IL-18 were provided in five studies and pooled SMD was 1.27 (95% CI: 0.64–1.90; *P* < 0.001), indicating elevated levels of IL-18 in peripheral blood were associated with psoriasis risk (Fig S3 in S1 File). Seven studies included data on the outcome index of IFN-γ; the SMD was 1.90 (95% CI: 1.27–2.52; *P* <0.001), again showing increased circulating levels of IFN-γ in psoriasis patients compared to the control groups (Fig S4 in S1 File). We found no apparent association between levels of IL-4, IL-12, IL-22, IL-23, IL-35, IL-36 and TGF-β in circulation and the risk of psoriasis. The pooled effect size for serum IL-4, IL-12, IL-22, IL-23, IL-35, IL-36, and TGF-β levels were: -0.35 (95% CI: -1.69–1.00; P = 0.612); 0.58 (95% CI: -0.15–1.32; P = 0.119), -0.07 (95% CI: -1.41–1.26; P = 0.913); -0.70 (95% CI: -1.95–0.55; P = 0.271); -2.60 (95% CI: -6.34–1.14; P = 0.172); 1.82 (95% CI: -0.56–4.20; P = 0.134); and -0.85 (95% CI:-2.45–0.76;P = 0.301), respectively.

#### Heterogeneity test for the association with circulating inflammatory elements and the risks of psoriasis

**Fig 2:**
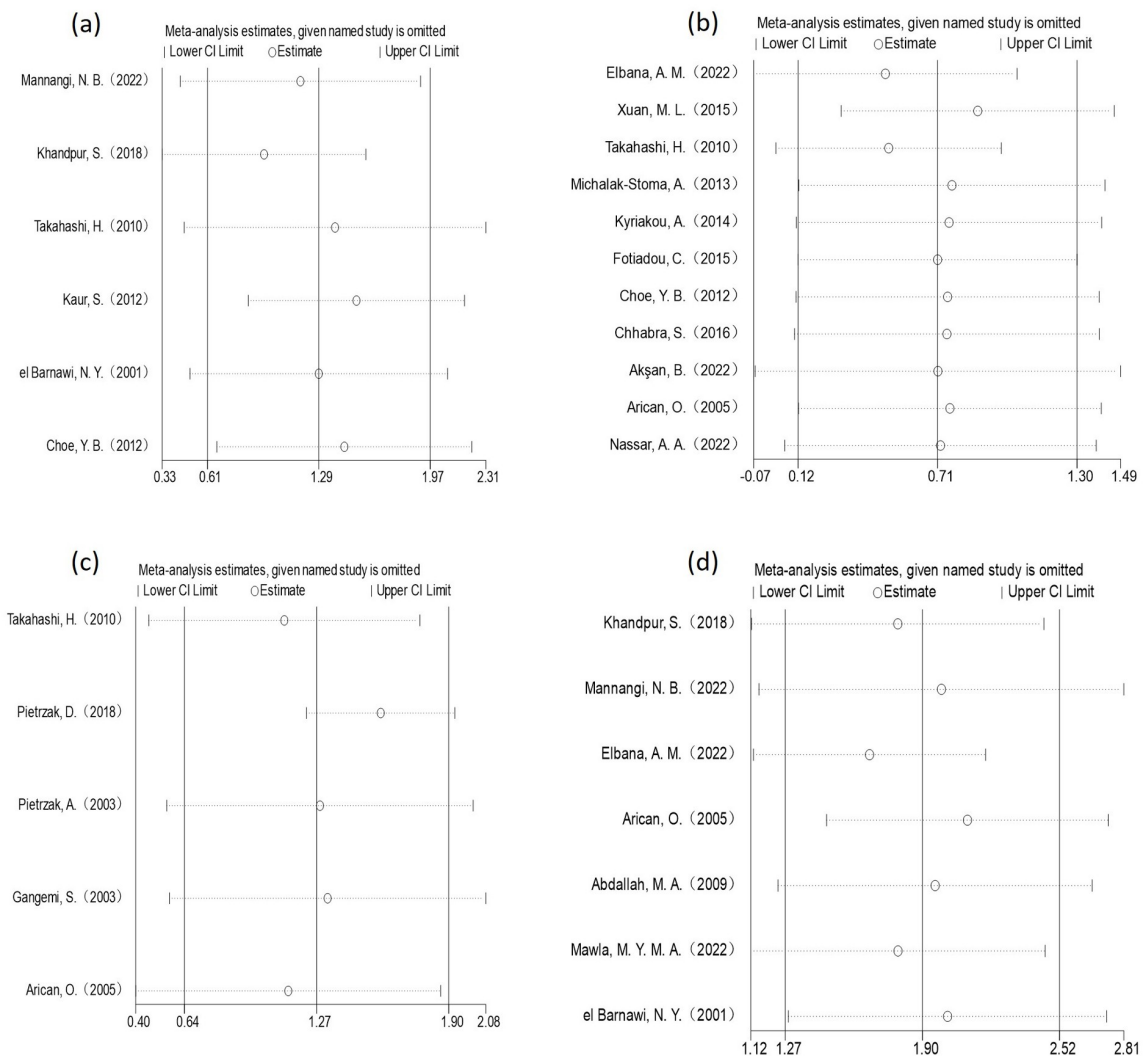
Sensitivity analysis of the association between circulating inflammatory factors and psoriasis risk. Sensitivity analysis of the correlation between IL-2 and the risk of psoriasis(a). Sensitivity analysis of the correlation between IL-17 and the risk of psoriasis(b). Sensitivity analysis of the correlation between IL-18 and the risk of psoriasis(c). Sensitivity analysis of the correlation between IFN-γ and the risk of psoriasis(d).

Since forest plots showed significant heterogeneity across all circulating inflammatory factors, meta-regression analysis was performed including age, sex, study type, number of patients groups (Cases nP), and severity of psoriasis (Table 3) to explore sources of heterogeneity. Subgroup analysis by region, measurement method, psoriasis type, and study type (Table 4) was employed to analyze sources of heterogeneity. The statistically significant and highly heterogeneous outcome indicators IL-2, 17, 18, and IFN-γ were subjected to sensitivity analysis to find the source of heterogeneity (Fig 2a–2d).

**Table 3:** Regression analysis of the association between circulating inflammatory cytokines and the risk of psoriasis.

Inflammmatory factors	Age		Sex		PASI		Study type		Cases nP	
	P>|t|	Coef [95% Coef.Interval]	P>|t|	Coef [95% Coef.Interval]	P>|t|	Coef [95% Coef.Interval]	P>|t|	Coef [95%Coef.Interval]	P>|t|	Coef [95%Coef.Interval]
**IL-2**	0.24	-0.16(-0.59,0.26)	0.48	0.06(-0.24, 0.36)	/	/	0.16	1.43(-0.88, 3.75)	0.60	-0.01(-0.05,0.03)
**IL-4**	0.91	0.02(-1.64, 1.68)	0.43	-0.13(-1.47, 1.21)	/	/	0.84	-0.42(-8.26, 7.41)	0.87	-0.03(-0.67,0.62)
**IL-12**	0.97	0.00(-0.44, 0.45)	0.98	-0.00(-0.10, 0.10)	/	/	-0.47	-0.34(-2.66, 1.98)	0.36	0.01(-0.02,0.04)
**IL-17**	0.03	0.21(0.03, 0.38)	0.05	-0.07(-0.14, 0.00)	0.36	-0.06(- 0.22, 0.11)	0.77	-0.28(-2.38, 1.83)	0.58	0.00(-0.01,0.02)
**IL-18**	0.49	0.04(-0.16, 0.24)	0.2	-0.03(-0.10, 0.04)	/	/	0.02	-1.08(-1.82, -0.34)	0.90	0.00(-0.02,0.02)
**IL-22**	0.58	-0.62(-10.68, 9.44)	0.99	-0.00(-0.87, 0.87)	/	/	0.10	-2.66(-6.42,1.11)	0.78	0.02(-0.23,0.27)
**IL-23**	0.79	0.10(-1.25, 1.44)	0.94	0.00(-0.19, 0.20)	/	/	0.64	0.93(-4.72,6.58)	0.10	-0.06(-0.14,0.02)
**TGF-β**	0.89	-0.05(-3.77, 3.67)	0.79	0.18(-6.31, 6.67)	/	/	0.58	1.80(-9.96,13.56)	0.87	0.01(-0.18,0.20)
**IFN-γ**	0.48	0.09(-0.35, 0.53)	0.77	0.03(-0.30, 0.36)	/	/	0.82	-0.18(-2.13,1.77)	0.83	-0.00(-0.04,0.03)

**Table 4:** Subgroup analysis of the association between circulating inflammatory cytokines and psoriasis risk.

Inflammatory factors	subgroup analysis	Number of studies	I^2^	P_h_	SMD(95%CI)	P-value
**IL-2**	Region					
	Asia	4	92.60%	0.000	1.575(0.767,2.384)	0.000
	Other continents	2	90.60%	0.001	0.711(-0.452,1.874)	0.231
	Method					
	ELISA	5	90.10%	0.000	1.516(0.854,2.177)	0.000
	Other methods	1	/	/	0.144(-0.221,-0.508)	0.44
	Types					
	Psoriasis vulgaris	1	/	/	1.332(0.717,1.947)	0.000
	Plaque psoriasis	1	/	/	0.144(-0.221,0.508)	0.44
	Other psoriasis types	4	92.60%	0.000	1.575(0.767,2.384)	0.000
	Study type 1	4	93.8%	0.000	0.864(0.134,1.594)	0.020
	Study type 2	2	92.3%	0.000	2.337(0.303,4.371)	0.024
**IL-4**	Region					
	Asia	3	93.50%	0.000	-0.862(-2.370,0.645)	0.262
	Other continents	1	/	/	1.178(0.576, 1.781)	0.000
	Method					
	ELISA	4	98.20%	0.000	-0.348(-1.691, 0.996)	0.612
	Other methods	0				
	Types					
	Psoriasis vulgaris	2	92.80%	0.000	0.298(-1.448, 2.045)	0.738
	Plaque psoriasis	0				
	Other psoriasis types	2	96.70%	0.000	-1.008(-3.613, 1.597)	0.448
	Study type 1	2	75.4%	0.044	-0.113(-0.999,0.773)	0.803
	Study type 2	2	97.9%	0.000	-0.579(-4.042,2.884)	0.743
**IL-12**	Region					
	Asia	2	81.30%	0.021	1.145(0.401, 1.888)	0.003
	Other continents	3	73.20%	0.024	0.181(-0.410,0.773)	0.548
	Method					
	ELISA	5	97.20%	0.000	0.585(-0.151,1.320)	0.119
	Other methods	0				
	Types					
	Psoriasis vulgaris					
	Plaque psoriasis	2	74.80%	0.046	0.435(-0.406,1.276)	0.311
	Other psoriasis types	3	94.90%	0.000	0.666(-0.434,1.766)	0.236
	Study type 1	3	94.8%	0.000	0.719(-0.447,1.886)	0.227
	Study type 2	2	69.3%	0.071	0.370(-0.303,1.042)	0.281
**IL-17**	Region					
	Asia	5	96.80%	0.001	0.520(-0.721,1.761)	0.412
	Other continents	5	92.50%	0.000	0.883(0.238,1.528)	0.007
	Method					
	ELISA	10	95.00%	0.000	0.713(0.123,1.304)	0.018
	Other methods	0				
	Types					
	Psoriasis vulgaris	0				
	Plaque psoriasis	5	0%	0.837	0.377(0.149,0.605)	0.001
	Other psoriasis types	5	97.5%	0.000	1.064(-0.018,2.145)	0.054
	Study type 1	8	96.0%	0.000	0.770(-0.096,1.636)	0.081
	Study type 2	2	60.9%	0.110	0.554(0.154,0.953)	0.007
**IL-18**	Region					
	Asia	2	0%	0.851	1.775(1.479,2.071)	0.000
	Other continents	3	29.80%	0.24	0.764(0.353,1.175)	0.000
	Method					
	ELISA	5	95.90%	0.000	1.269(0.637,1.901)	0.000
	Other methods	0				
	Types					
	Psoriasis vulgaris	0				
	Plaque psoriasis	1	/	/	1.830(1.181,2.479)	0.000
	Other psoriasis types	4	88.60%	0.000	1.133(0.404,1.862)	0.002
	Study type 1	2	0.0%	0.851	1.775(1.479,2.071)	0.000
	Study type 2	3	29.8%	0.240	0.764(0.353,1.175)	0.000
**IL-22**	Region					
	Asia	1	/	/	-2.069(-2.722,-1.416)	0.000
	Other continents	3	81.6	0.004	0.587(-0.181,1.354)	0.134
	Method					
	ELISA	4	98.50%	0.000	-0.075(-1.412,1.263)	0.913
	Other methods	0				
	Types					
	Psoriasis vulgaris	1	/	/	1.348(0.731,1.964)	0.000
	Plaque psoriasis	1	/	/	-0.168(-0.828,0.492)	0.618
	Other psoriasis types	2	97.60%	0.000	-0.743(-3.320,1.834)	0.572
	Study type 1	3	81.6%	0.004	0.587(-0.181,1.354)	0.134
	Study type 2	1	/	/	-2.069(-2.722,-1.416)	0.000
**IL-23**	Region					
	Asia	2	98.80%	0.000	-1.549(-4.840,1.742)	0.356
	Other continents	3	43.60%	0.17	-0.170(-0.571,0.231)	0.406
	Method					
	ELISA	5	98.80%	0.000	-0.702(-1.953,0.548)	0.271
	Other methods	0				
	Types					
	Psoriasis vulgaris	2	0%	0.9	0.109(-0.300,0.519)	0.601
	Plaque psoriasis	1	/	/	0.040(-0.450,0.530)	0.871
	Other psoriasis types	2	98.40%	0.000	-1.869(-4.526,0.788)	0.618
	Study type 1	4	97.1%	0.000	-0.888(-2.441,0.665)	0.262
	Study type 2	1	/	/	0.040(-0.450,0.530)	0.871
**TGF-β**	Region					
	Asia	2	88.50%	0.003	0.020(-0.868,0.907)	0.964
	Other continents	2	98.50%	0.000	-1.763(-6.173,2.647)	0.433
	Method					
	ELISA	4	99.10%	0.000	-0.846(-2.447,0.755)	0.301
	Other methods	0				
	Types					
	Psoriasis vulgaris	3	81.60%	0.004	0.147(-0.539,0.832)	0.675
	Plaque psoriasis	0				
	Other psoriasis types	1	/	/	-4.014(-4.781,-3.246)	0.000
	Study type 1	3	97.6%	0.000	-1.304(-3.577,0.968)	0.261
	Study type 2	1	/	/	0.492(-0.005,0.989)	0.052
**IFN-γ**	Region					
	Asia	3	86.40%	0.001	1.556(0.715–2.397)	0.000
	Other continents	4	86.60%	0.000	2.158(1.222–3.094)	0.000
	Method					
	ELISA	7	87.30%	0.000	1.897(1.275,2.519)	0.000
	Other methods	0				
	Types					
	Psoriasis vulgaris	1	/	/	1.252(0.644,1.860)	0.000
	Plaque psoriasis	1	/	/	0.708(0.147,1.268)	0.013
	Other psoriasis types	5	86.60%	0.000	2.281(1.532,3.030)	0.000
	Study type 1	3	72.0%	0.028	1.780(0.983,2.577)	0.000
	Study type 2	4	92.5%	0.000	1.981(1.009,2.953)	0.000

For regression analysis based on age, sex, study type, psoriasis severity, and number of psoriasis patients, the age of IL-17 was P = 0.03<0.05, suggesting that age may be a significant source of heterogeneity in IL-17 serum levels. The study type of IL-18 P = 0.02<0.05, indicating that the study type may be a significant source of heterogeneity in serum levels of IL-18. In the analysis of the other variables, the P values were greater than 0.05, and none of them reduced heterogeneity.

Based on subgroup analysis of psoriasis types, study types, regions, and laboratory measurements, we know that IL-17 has P = 0.001<0.05 for plaque psoriasis and P = 0.054 >0.05 for other types of psoriasis, indicating that the serum level of IL-17 in plaque psoriasis is more different than other types of psoriasis. As expected, psoriasis types further reduce heterogeneity because plaque types have higher serum concentrations compared to other types. The P values for IL-18 in Asia and elsewhere, study type 1 and study type 2 were all 0.000, indicating that there was no significant difference in serum levels of IL-18 between study types and regions. The remaining negative results did not explain the high level of heterogeneity between studies (all I^2^ >50%). These suggest that the type of study in the article had no significant effect on SMD for any of the inflammatory markers studied, nor did they differ significantly between studies.

**Fig 3:**
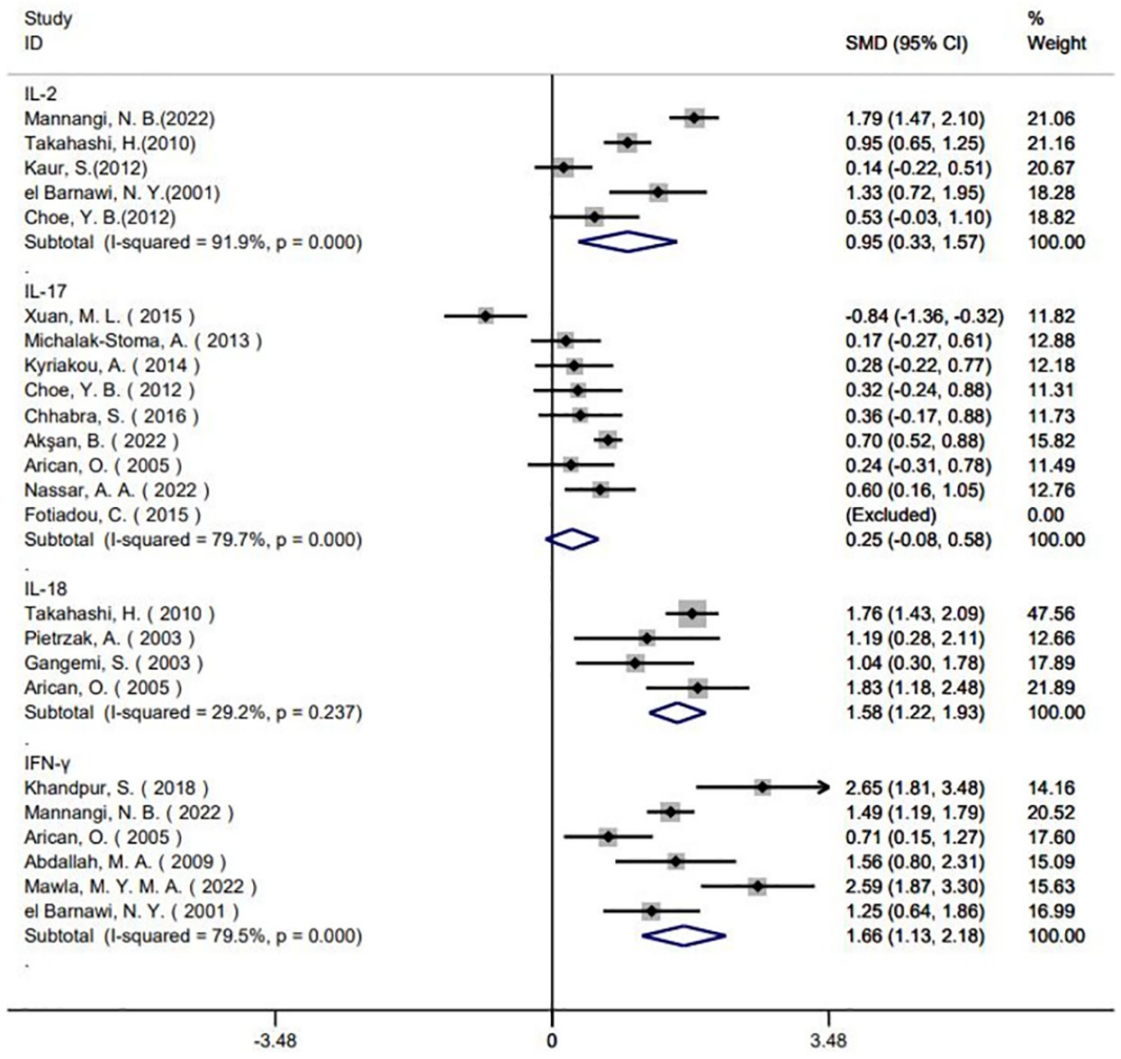
Forest plot of publication bias test for the correlation between IL-2, IL-17, IL-18, IFN-γ and the risk of psoriasis after removing studies that may cause heterogeneity.

The sensitivity analysis based on literature sources showed that Khandpur (2018) was the primary source of IL-2 heterogeneity, Elbana (2022) and Takahashi (2010) were the primary sources of IL-17 heterogeneity, Pietrzak (2018) was the main source of IL-18 heterogeneity, and Elbana (2022) was the main source of IFN-γ heterogeneity. Removing these sources of heterogeneity gives the forest plot shown in Fig 3. We conclude that Pietrzak (2018) is the main source of heterogeneity of the inflammatory factor IL-18, but for IL-2, IL-17 and IFN-γ significant heterogeneity remained.

#### Publication bias test for the association of circulating inflammatory factors with the risk of psoriasis

A meta-analysis of the association of expression levels for each inflammatory factor with the risks of psoriasis was performed to check for bias. The funnel plot and Egger test results (all *P*-values >0.05 for all factors) do not indicate publication bias (Table 5).

**Table 5:** Egger’s results of bias analysis of the association between circulating inflammatory factors and psoriasis risk.

index	study	P>|t|	[95% Conf.Interval]	
**IL-2**	6	0.586	-10.46157	16.11826
**IL-4**	4	0.241	-58.09131	25.89655
**IL-18**	5	0.809	-10.58513	12.49414
**IL-12**	5	0.43	-25.25196	14.02665
**IL-17**	10	0.826	-8.09325	6.621735
**IL-22**	5	0.459	-49.16406	28.48783
**IL-23**	6	0.762	-48.58001	61.43118
**IFN-γ**	7	0.319	-4.189436	10.51678
**TGF-β**	4	0.629	-49.08086	37.67439

The main limitations of this study were significant heterogeneity between the included studies and the authors’ different methods of expressing effect sizes. We therefore investigated publication bias to look for sources of heterogeneity. In addition, we performed sensitivity analyses, subgroup analyses, and regression analyses to locate studies that differed significantly from other studies. Finally, the age of IL-17 and the classification parameters of IL-18 study type and region can explain heterogeneity. Despite all these efforts to account for heterogeneity, heterogeneity for most inflammatory factors remained high in studies. The main reasons for this question remain differences in design and quality, measurement methods, measurement of outcome indicators, and the genetic background of participants in these studies.

## Discussion

In the complex immune system, inflammatory cytokines and their receptors constitute a comprehensive regulatory framework that can exert significant influence in the onset and progression of disease [66–68]. Inflammatory factors can thus be a crucial part of diagnosis, prognosis and therapy in autoimmune diseases [69, 70]. In psoriasis, however, the relevant biomarkers remain unclear. This meta-analysis summarized and analyzed data from 57 studies to explore the association between circulating inflammatory factors and psoriasis risk. Elevated circulating levels of the inflammatory cytokines IL-2, IL-17, IL-18 and IFN-γ were statistically significantly correlated with the risk of psoriasis, while no significant correlation was found for the levels of circulating inflammatory factors IL-4, IL-12, IL-22, IL-23, IL-35, IL-36 and TGF-β.

IL-2 is a cell growth factor with various immunoregulatory functions and biological characteristics, secreted mostly by activated helper T lymphocytes [71, 72]. Elevated serum IL-2 levels are associated with significant disease progression in many autoimmune diseases and cancers, including melanoma, autoimmune hepatitis, and systemic lupus erythematosus [73, 74]. Our findings suggest that psoriasis is associated with higher IL-2 levels, possibly because IL-2 is engaged in its initiation and development [75, 76].

The role of IL-17 in autoimmune diseases has gained interest in recent years. IL-17 is mostly generated by activated Th17, which can, directly and indirectly, induce various cells to produce inflammatory cytokines and chemokines that mediate inflammatory responses [8]. Clinical studies have demonstrated that anti-IL-17 biologics (such as secukinumab and ixekizumab) can have a good curative effect on autoimmune diseases [77]. Our meta-analysis results suggest that IL-17 is linked to the risk of psoriasis and indicate that IL-17 is likely to be a biomarker for psoriasis in patients.

Previous studies have demonstrated elevated levels of circulating factor IL-18 in the serum of individuals with psoriasis. IL-18 is a complex inflammatory factor with multiple immunomodulatory effects [78]. In its own immune regulation, IL-18 is a cytokine secreted by helper T cell 1 (Th1) that then induces Th1 cells and NK cells to secrete IL-2, IFN-γ and other inflammatory factors, inhibits the production of IL-4 and IL-10, and promotes the proliferation of Th1 cells [79]. IL-18 is also implicated in the modulation of Th2 cytokines and inflammatory agents, as are IL-4 and IL-13 [54]. Further, IL-18 can be secreted by keratinocytes and is involved in psoriasis development [80], which is in line with our findings. Therefore, the function of IL-18 in psoriasis development is likely important.

IFN-γ is associated with a variety of autoimmune diseases in humans, ranging from psoriasis to systemic lupus erythematosus to rheumatoid arthritis [24]. In the pathogenesis of psoriasis, IFN-γ is the primary circulating inflammatory factor produced by Th1 cells, which inhibits the differentiation of helper T cells Th0 and Th1 into Th2 cells [25]. IFN-γ mediates the contact between T cells and keratinocytes, promotes the migration of T cells to the lesion epidermis, and promotes the proliferation of keratinocytes through the expression of anti-apoptotic proteins in psoriatic skin tissues, thereby participating in psoriasis disease pathogenesis [26, 81]. Together with our meta-analysis, these observations suggest IFN-γ may be a useful biomarker for evaluating the risk of psoriasis.

The results of this meta-analysis suggest that circulating levels of IL-2, IL-17, IL-18, and IFN-γ may serve as biomarkers of psoriasis risk. A large number of clinical and experimental studies have shown that modulated biologic drugs that block inflammatory mediators are more effective in controlling disease and reducing the risk of adverse disease in patients compared with broad-spectrum immunosuppressants. Therefore, more and more researchers are actively looking for possible biomarkers of psoriasis for treatment and diagnosis, providing more reliable strategies for the treatment of psoriasis. Existing experimental studies have shown a very significant correlation between IL-2 cytokine levels and autoimmune diseases, especially psoriasis [15, 82] Therefore, therapies based on IL-2 regulatory T cells have been proposed. There are also many cytokines that have been shown to be associated with psoriasis, such as IL-17, which is considered to be the main pathogenic regulator in the regulation of activated T cells [8]. In the current clinical application, IL-17 inhibitors (e.g., sekukizumab, eccilizumab) have a good effect on psoriasis. IL-18 has also been found to be the initiator of the cytokine cascade, and the concentration of IL-18 in serum correlates with the severity index (PASI) of psoriasis [22, 83]. Recent data suggest that IFN-γ can sustain the progression of skin diseases [84]. Moreover, it has been proposed that the IL-23/IL-17 axis, as the central mechanism of the pro-inflammatory cycle of psoriasis, may transform into a major driver of disease caused by Th1 cells and IFN-γ [85]. The evidence provided by this meta-analysis suggests that these inflammatory factors are involved in the progression of psoriasis and play an important role in it. Therefore, the results of this analysis are expected to provide a more reliable strategy for the treatment and diagnosis of targeted circulating inflammatory cytokines for psoriasis.

We do not see a statistically meaningful correlation between circulating levels of IL-4, IL-12, IL-22, IL-23, IL-35, IL-36 and TGF-β with the development of psoriasis. This result was unexpected as these circulating inflammatory factors are thought to play a part in the psoriasis cascade, which suggests their levels in psoriasis patients should deviate from normal levels. However, in our sample collections, we have observed that the degree of change in circulating inflammatory factors is often not obvious, and we suggest this is the reason for our results. Second, the amount of circulating inflammatory cytokines released is not necessarily proportional to their function. Furthermore, the finding may be attributed to the limited availability of included research on circulating inflammatory factors and the low sample sizes of individual studies. Thus, additional research with larger sample sizes is warranted to better assess the role of these cytokines and elaborate on the distinct pathogenesis of psoriasis.

Several aspects of this meta-analysis are limited and deserve additional consideration. First, the regions, populations and psoriasis types covered in this research are restricted. Second, the kits and manufacturing units used to measure inflammatory factors varied among the enrolled studies, which may add to the heterogeneity among studies. Finally, we performed preliminary sub-group analyses of the risks for various types of psoriasis but were unable to reach more comprehensive conclusions due to incomplete data in the original studies. Lastly, despite the regression and subgroup analyses we performed to investigate potential sources of heterogeneity, inflammatory factors remained highly heterogeneous.

## Key message

Overall, our meta-analysis suggests that circulating inflammatory cytokine IL-2, IL-17, IL-18 and IFN-γ levels may be potent biomarkers of psoriasis in patients. Results indicate that elevated levels of circulating inflammatory cytokines IL-2, IL-17, IL-18, and IFN-γ are related to enhanced risk of psoriasis and may help to predict clinical outcomes in individuals with psoriasis. Moreover, based on the regression and subgroup analysis results of different psoriasis subtypes, different inflammatory factors may be involved in different types of psoriasis. Studying this question in depth requires research with larger sample sizes from a broad geographic and cultural area and for different types of psoriasis. Given the relatively simple method of blood sample collection and measurement, we suggest that testing of circulating inflammatory factors should be done on a broad scale and the data used for intensive research on the mechanistic action of cytokines in autoimmune diseases. This may provide new insights into ways to reduce the risk of psoriasis and offer additional indicators helpful for assessing prognosis in psoriasis patients.

### Statement of ethics

The report was carried out in adherence with the Preferred Reporting Items for Systematic Reviews and Meta-Analyses (PRISMA) group standards for reporting meta-analysis of observational studies.

## Supporting information

S1 File (DOC)

S2 File (DOC)
