# Supplementary material for: Circulating inflammatory cytokines and psoriasis risk: A systematic review and meta-analysis
Source: PLoS One. 2023 Oct 26;18(10):e0293327. doi: 10.1371/journal.pone.0293327 (PMC10602280; doi:10.1371/journal.pone.0293327)
Supplement: S1 File — (DOC) [file pone.0293327.s001.doc]

# Supplementary Material for:

**Circulating Inflammatory Cytokines and Psoriasis Risk: A Systematic Review and Meta-Analysis**

**Authors:** Xiao-Qing Liu, Pei-Lin Zhou, Xin-Yu Yin, Ai-Xue Wang, Da-Hu Wang, Yun Yang, Qiang Liu

**Table S1** search strategy for the present meta-analysis

| **Number** | **Search** | **Results** |
| --- | --- | --- |
| **1** | "Psoriasis"[MeSH Terms] | 46,900 |
| **2** | "Psoriasis"[Title/Abstract] OR "Psoriases"[Title/Abstract] OR ("Pustulosis"[All Fields] AND "of palms"[Title/Abstract] AND "Soles"[Title/Abstract]) OR "pustulosis palmaris et plantaris"[Title/Abstract] OR "palmoplantaris pustulosis"[Title/Abstract] OR ("pustular psoriasis of palms"[Title/Abstract] AND "Soles"[Title/Abstract]) | 48,770 |
| **3** | #1 OR #2 | 60,638 |
| **4** | "Case-Control Studies"[MeSH Terms] | [1,385,393](https://pubmed.ncbi.nlm.nih.gov/?sort=date&term="Case-Control+Studies"%5BMesh%5D&size=200) |
| **5** | "case control studies"[Title/Abstract] OR "case control study"[Title/Abstract] OR "studies case control"[Title/Abstract] OR "study case control"[Title/Abstract] OR "case comparison studies"[Title/Abstract] OR "case comparison studies"[Title/Abstract] OR "case comparison study"[Title/Abstract] OR (("Studies"[All Fields] OR "Study"[All Fields] OR "study s"[All Fields] OR "studying"[All Fields] OR "studys"[All Fields]) AND "Case-Comparison"[Title/Abstract]) OR (("Studies"[All Fields] OR "Study"[All Fields] OR "study s"[All Fields] OR "studying"[All Fields] OR "studys"[All Fields]) AND "Case-Comparison"[Title/Abstract]) OR "case compeer studies"[Title/Abstract] OR (("Studies"[All Fields] OR "Study"[All Fields] OR "study s"[All Fields] OR "studying"[All Fields] OR "studys"[All Fields]) AND "Case-Compeer"[Title/Abstract]) OR ((("ieee int conf automation sci eng case"[Journal] OR "case phila"[Journal] OR "Case"[All Fields]) AND "Referrent"[All Fields]) AND "Studies"[Title/Abstract]) OR ((("ieee int conf automation sci eng case"[Journal] OR "case phila"[Journal] OR "Case"[All Fields]) AND "Referrent"[All Fields]) AND "Studies"[Title/Abstract]) OR ((("ieee int conf automation sci eng case"[Journal] OR "case phila"[Journal] OR "Case"[All Fields]) AND "Referrent"[All Fields]) AND "Study"[Title/Abstract]) OR ((("Studies"[All Fields] OR "Study"[All Fields] OR "study s"[All Fields] OR "studying"[All Fields] OR "studys"[All Fields]) AND ("ieee int conf automation sci eng case"[Journal] OR "case phila"[Journal] OR "Case"[All Fields])) AND "Referrent"[Title/Abstract]) OR ((("Studies"[All Fields] OR "Study"[All Fields] OR "study s"[All Fields] OR "studying"[All Fields] OR "studys"[All Fields]) AND ("ieee int conf automation sci eng case"[Journal] OR "case phila"[Journal] OR "Case"[All Fields])) AND "Referrent"[Title/Abstract]) OR "case referent studies"[Title/Abstract] OR "case referent studies"[Title/Abstract] OR "case referent study"[Title/Abstract] OR "studies case referent"[Title/Abstract] OR "study case referent"[Title/Abstract] OR "case base studies"[Title/Abstract] OR "case base studies"[Title/Abstract] OR "studies case base"[Title/Abstract] OR "case control studies"[Title/Abstract] OR "case control study"[Title/Abstract] OR "studies case control"[Title/Abstract] OR "study case control"[Title/Abstract] OR "nested case control studies"[Title/Abstract] OR "case control studies nested"[Title/Abstract] OR "case control study nested"[Title/Abstract] OR "nested case control studies"[Title/Abstract] OR "nested case control study"[Title/Abstract] OR "studies nested case control"[Title/Abstract] OR "study nested case control"[Title/Abstract] OR "matched case control studies"[Title/Abstract] OR "case control studies matched"[Title/Abstract] OR "case control study matched"[Title/Abstract] OR "matched case control studies"[Title/Abstract] OR "matched case control study"[Title/Abstract] OR "studies matched case control"[Title/Abstract] OR "study matched case control"[Title/Abstract] | 127,061 |
| **6** | #4 OR #5 | [1,422,437](https://pubmed.ncbi.nlm.nih.gov/?term=%234+OR+%235&sort=&size=200) |
| **7** | "Cross-Sectional Studies"[MeSH Terms] | 453,945 |
| **8** | "cross sectional studies"[Title/Abstract] OR "cross sectional studies"[Title/Abstract] OR "cross sectional study"[Title/Abstract] OR "studies cross sectional"[Title/Abstract] OR "study cross sectional"[Title/Abstract] OR "cross sectional analysis"[Title/Abstract] OR "analyses cross sectional"[Title/Abstract] OR "cross sectional analyses"[Title/Abstract] OR "disease frequency surveys"[Title/Abstract] OR "cross sectional survey"[Title/Abstract] OR "cross sectional survey"[Title/Abstract] OR "cross sectional surveys"[Title/Abstract] OR "survey cross sectional"[Title/Abstract] OR "surveys cross sectional"[Title/Abstract] OR (("survey s"[All Fields] OR "surveyed"[All Fields] OR "surveying"[All Fields] OR "surveys and questionnaires"[MeSH Terms] OR ("Surveys"[All Fields] AND "questionnaires"[All Fields]) OR "surveys and questionnaires"[All Fields] OR "Survey"[All Fields] OR "Surveys"[All Fields]) AND "disease frequency"[Title/Abstract]) OR (("Disease"[MeSH Terms] OR "Disease"[All Fields] OR "diseases"[All Fields] OR "disease s"[All Fields] OR "diseased"[All Fields]) AND "frequency survey"[Title/Abstract]) OR (("survey s"[All Fields] OR "surveyed"[All Fields] OR "surveying"[All Fields] OR "surveys and questionnaires"[MeSH Terms] OR ("Surveys"[All Fields] AND "questionnaires"[All Fields]) OR "surveys and questionnaires"[All Fields] OR "Survey"[All Fields] OR "Surveys"[All Fields]) AND "disease frequency"[Title/Abstract]) OR "analysis cross sectional"[Title/Abstract] OR "analyses cross sectional"[Title/Abstract] OR "analysis cross sectional"[Title/Abstract] OR "cross sectional analyses"[Title/Abstract] OR "cross sectional analysis"[Title/Abstract] OR "prevalence studies"[Title/Abstract] OR "prevalence study"[Title/Abstract] OR "studies prevalence"[Title/Abstract] OR "study prevalence"[Title/Abstract] | 320,451 |
| **9** | #7 OR #8 | 549,118 |
| **10** | "Cohort Studies"[MeSH Terms] | [2,439,141](https://pubmed.ncbi.nlm.nih.gov/?sort=date&term="Cohort+Studies"%5BMesh%5D&size=200) |
| **11** | "cohort studies"[Title/Abstract] OR "cohort study"[Title/Abstract] OR "studies cohort"[Title/Abstract] OR "study cohort"[Title/Abstract] OR "concurrent studies"[Title/Abstract] OR "studies concurrent"[Title/Abstract] OR "concurrent study"[Title/Abstract] OR "study concurrent"[Title/Abstract] OR "closed cohort studies"[Title/Abstract] OR (("Cohort"[All Fields] OR "cohort s"[All Fields] OR "cohorte"[All Fields] OR "cohorts"[All Fields]) AND "studies closed"[Title/Abstract]) OR "closed cohort study"[Title/Abstract] OR (("Cohort"[All Fields] OR "cohort s"[All Fields] OR "cohorte"[All Fields] OR "cohorts"[All Fields]) AND "study closed"[Title/Abstract]) OR (("Studies"[All Fields] OR "Study"[All Fields] OR "study s"[All Fields] OR "studying"[All Fields] OR "studys"[All Fields]) AND "closed cohort"[Title/Abstract]) OR (("Studies"[All Fields] OR "Study"[All Fields] OR "study s"[All Fields] OR "studying"[All Fields] OR "studys"[All Fields]) AND "closed cohort"[Title/Abstract]) OR "birth cohort studies"[Title/Abstract] OR "birth cohort study"[Title/Abstract] OR (("Cohort"[All Fields] OR "cohort s"[All Fields] OR "cohorte"[All Fields] OR "cohorts"[All Fields]) AND "studies birth"[Title/Abstract]) OR "cohort study birth"[Title/Abstract] OR "studies birth cohort"[Title/Abstract] OR "study birth cohort"[Title/Abstract] OR "analysis cohort"[Title/Abstract] OR "analyses cohort"[Title/Abstract] OR "cohort analyses"[Title/Abstract] OR "cohort analysis"[Title/Abstract] OR "historical cohort studies"[Title/Abstract] OR (("Cohort"[All Fields] OR "cohort s"[All Fields] OR "cohorte"[All Fields] OR "cohorts"[All Fields]) AND "studies historical"[Title/Abstract]) OR "cohort study historical"[Title/Abstract] OR "historical cohort study"[Title/Abstract] OR "study historical cohort"[Title/Abstract] OR "studies historical cohort"[Title/Abstract] OR "incidence studies"[Title/Abstract] OR "incidence study"[Title/Abstract] OR "studies incidence"[Title/Abstract] OR "study incidence"[Title/Abstract] | 332,195 |
| **12** | #10 OR #11 | [2,532,120](https://pubmed.ncbi.nlm.nih.gov/?term=%2310+OR+%2311&sort=&size=200) |
| **13** | #6 OR #9 OR #12 | [3,245,352](https://pubmed.ncbi.nlm.nih.gov/?term=%236+OR+%239+OR+%2312&sort=&size=200) |
| **14** | "Interleukin-12"[MeSH Terms] | 15,173 |
| **15** | "Interleukin-12"[Title/Abstract] OR "natural killer cell stimulatory factor"[Title/Abstract] OR "IL-12"[Title/Abstract] OR "cytotoxic lymphocyte maturation factor"[Title/Abstract] OR "IL-12"[Title/Abstract] OR "il 12 p70"[Title/Abstract] OR "interleukin 12 p70"[Title/Abstract] OR "interleukin 12 p70"[Title/Abstract] OR "Interleukin-12"[Title/Abstract] OR "IL12"[Title/Abstract] OR "edodekin alfa"[Title/Abstract] | 29,339 |
| **16** | #14 OR #15 | 31,976 |
| **17** | #3 AND #13 AND #16 | 161 |
| **18** | "Interleukin-17"[MeSH Terms] | 14,174 |
| **19** | "Interleukin-17"[Title/Abstract] OR "Interleukin-17"[Title/Abstract] OR "CTLA-8"[Title/Abstract] OR "IL-17"[Title/Abstract] OR "Interleukin-17F"[Title/Abstract] OR "Interleukin-17F"[Title/Abstract] OR "IL-17F"[Title/Abstract] OR "cytokine ml 1"[Title/Abstract] OR "cytokine ml 1"[Title/Abstract] OR "Interleukin-17C"[Title/Abstract] OR "Interleukin-17C"[Title/Abstract] OR "IL-17C"[Title/Abstract] OR (("cytokin"[All Fields] OR "cytokine s"[All Fields] OR "cytokines"[MeSH Terms] OR "cytokines"[All Fields] OR "Cytokine"[All Fields] OR "cytokinic"[All Fields] OR "cytokins"[All Fields]) AND "CX2"[Title/Abstract]) OR ("CX2"[All Fields] AND "Cytokine"[Title/Abstract]) OR "Interleukin-17E"[Title/Abstract] OR "Interleukin-17E"[Title/Abstract] OR "Interleukin-25"[Title/Abstract] OR "Interleukin-25"[Title/Abstract] OR "IL-17E"[Title/Abstract] OR "IL-17E"[Title/Abstract] OR "Interleukin-17A"[Title/Abstract] OR "Interleukin-17A"[Title/Abstract] OR "cytotoxic t lymphocyte associated antigen 8"[Title/Abstract] OR "cytotoxic t lymphocyte associated antigen 8"[Title/Abstract] OR "IL-17A"[Title/Abstract] OR "CTLA8"[Title/Abstract] OR "Interleukin-17B"[Title/Abstract] OR "Interleukin-17B"[Title/Abstract] OR "IL-17B"[Title/Abstract] | 30,841 |
| **20** | #18 OR #19 | 32,460 |
| **21** | #3 AND #13 AND #20 | 362 |
| **22** | "interleukin-22"[Title/Abstract] OR "IL-22"[Title/Abstract] OR "il 10 related t cell derived inducible factor"[Title/Abstract] OR (("interleukin-22"[Supplementary Concept] OR "interleukin-22"[All Fields] OR "interleukin-22"[All Fields]) AND "mouse"[Title/Abstract]) OR (("interleukin-22"[Supplementary Concept] OR "interleukin-22"[All Fields] OR "IL-22"[All Fields]) AND "protein mouse"[Title/Abstract]) OR ("IL22"[All Fields] AND "protein human"[Title/Abstract]) OR (("interleukin-22"[Supplementary Concept] OR "interleukin-22"[All Fields] OR "interleukin-22"[All Fields]) AND "protein human"[Title/Abstract]) | 4,786 |
|  |  |  |
|  |  |  |
| **23** | #3 AND #13 AND #22 | 50 |
| **24** | "Interleukin-23"[MeSH Terms] | 4,663 |
| **25** | "Interleukin-23"[Title/Abstract] OR "Interleukin-23"[Title/Abstract] OR "IL-23"[Title/Abstract] | 7,463 |
| **26** | #24 OR #25 | 9,195 |
| **27** | #3 AND #13 AND #26 | 204 |
| **28** | "interleukin-35"[Title/Abstract] OR "interleukin-35"[Title/Abstract] OR "IL-35"[Title/Abstract] OR ("IL-35"[All Fields] AND "protein human"[Title/Abstract]) | 906 |
| **29** | #3 AND #13 AND #28 | 2 |
| **30** | "interleukin-36"[Title/Abstract] OR "interleukin-36"[Title/Abstract] OR "IL-36"[Title/Abstract] OR ("IL-36"[All Fields] AND "protein human"[Title/Abstract]) | 553 |
| **31** | #3 AND #13 AND #30 | 16 |
| **32** | "Interferon-gamma"[MeSH Terms] | 71,012 |
| **33** | "Interferon-gamma"[Title/Abstract] OR "gamma-Interferon"[Title/Abstract] OR "interferon immune"[Title/Abstract] OR "immune interferon"[Title/Abstract] OR "type ii interferon"[Title/Abstract] OR "interferon type ii"[Title/Abstract] OR "interferon type ii"[Title/Abstract] OR "Interferon-gamma"[Title/Abstract] | 65,215 |
| **34** | #32 OR #33 | 103,019 |
| **35** | #3 AND #13 AND #34 | 94 |
| **36** | "Transforming Growth Factor beta"[MeSH Terms] | 68,176 |
| **37** | "transforming growth factor beta"[Title/Abstract] OR "milk growth factor"[Title/Abstract] OR (("Factor"[All Fields] OR "factor s"[All Fields] OR "factors"[All Fields]) AND "milk growth"[Title/Abstract]) OR (("growth and development"[MeSH Subheading] OR ("Growth"[All Fields] AND "development"[All Fields]) OR "growth and development"[All Fields] OR "Growth"[All Fields] OR "Growth"[MeSH Terms] OR "growths"[All Fields]) AND "factor milk"[Title/Abstract]) OR "TGF-beta"[Title/Abstract] OR "TGFbeta"[Title/Abstract] OR "platelet transforming growth factor"[Title/Abstract] OR "bone derived transforming growth factor"[Title/Abstract] OR "bone derived transforming growth factor"[Title/Abstract] | 84,822 |
| **38** | #36 OR #37 | 110,555 |
| **39** | #3 AND #13 AND #38 | 21 |
| **40** | "Interleukin-2"[MeSH Terms] | 39,853 |
| **41** | "Interleukin-2"[Title/Abstract] OR "Interleukin-2"[Title/Abstract] OR "IL-2"[Title/Abstract] OR "IL2"[Title/Abstract] OR "TCGF"[Title/Abstract] OR "interleukine 2"[Title/Abstract] OR "lymphocyte mitogenic factor"[Title/Abstract] OR "mitogenic factor lymphocyte"[Title/Abstract] OR "t cell growth factor"[Title/Abstract] OR "t cell growth factor"[Title/Abstract] OR "t cell stimulating factor"[Title/Abstract] OR "t cell stimulating factor"[Title/Abstract] OR "thymocyte stimulating factor"[Title/Abstract] OR "interleukin ii"[Title/Abstract] OR ((("res outreach"[Journal] OR "ro"[All Fields]) AND "23"[All Fields]) AND "6019"[Title/Abstract]) OR "RU-49637"[Title/Abstract] OR "RU-49637"[Title/Abstract] OR "RU49637"[Title/Abstract] | 75,958 |
| **42** | #40 OR #41 | 82,293 |
| **43** | #3 AND #13 AND #42 | 37 |
| **44** | "Interleukin-4"[MeSH Terms] | 24,204 |
| **45** | "Interleukin-4"[Title/Abstract] OR "Interleukin-4"[Title/Abstract] OR "b cell growth factor 1"[Title/Abstract] OR "b cell growth factor 1"[Title/Abstract] OR "b cell growth factor i"[Title/Abstract] OR "b cell growth factor i"[Title/Abstract] OR (("b lymphocytes"[MeSH Terms] OR "b lymphocytes"[All Fields] OR "B-Cell"[All Fields]) AND "proliferating factor"[Title/Abstract]) OR (("b lymphocytes"[MeSH Terms] OR "b lymphocytes"[All Fields] OR "B-Cell"[All Fields]) AND "proliferating factor"[Title/Abstract]) OR "b cell stimulating factor 1"[Title/Abstract] OR "b cell stimulating factor 1"[Title/Abstract] OR "b cell stimulatory factor 1"[Title/Abstract] OR "b cell stimulatory factor 1"[Title/Abstract] OR "BCGF-1"[Title/Abstract] OR "Binetrakin"[Title/Abstract] OR "BSF-1"[Title/Abstract] OR "IL-4"[Title/Abstract] OR "IL4"[Title/Abstract] OR (("mast cells"[MeSH Terms] OR ("mast"[All Fields] AND "cells"[All Fields]) OR "mast cells"[All Fields] OR ("mast"[All Fields] AND "Cell"[All Fields]) OR "mast cell"[All Fields]) AND "growth factor 2"[Title/Abstract]) OR (("mast cells"[MeSH Terms] OR ("mast"[All Fields] AND "cells"[All Fields]) OR "mast cells"[All Fields] OR ("mast"[All Fields] AND "Cell"[All Fields]) OR "mast cell"[All Fields]) AND "growth factor 2"[Title/Abstract]) OR ("MCGF"[All Fields] AND "2"[Title/Abstract]) OR "b cell stimulatory factor 1"[Title/Abstract] OR "b cell stimulatory factor 1"[Title/Abstract] | 56,426 |
| **46** | #44 OR #45 | 59,807 |
| **47** | #3 AND #13 AND #46 | 43 |
| **48** | "Interleukin-18"[MeSH Terms] | 6,715 |
| **49** | "Interleukin-18"[Title/Abstract] OR "Interleukin-18"[Title/Abstract] OR "ifn gamma inducing factor"[Title/Abstract] OR "IL-18"[Title/Abstract] OR "interferon gamma inducing factor"[Title/Abstract] OR "inducing factor interferon gamma"[Title/Abstract] OR "interferon gamma inducing factor"[Title/Abstract] OR "interferon gamma inducing factor"[Title/Abstract] OR "ifn gamma inducing factor"[Title/Abstract] OR "ifn gamma inducing factor"[Title/Abstract] OR (("induce"[All Fields] OR "induced"[All Fields] OR "inducer"[All Fields] OR "inducers"[All Fields] OR "induces"[All Fields] OR "inducibilities"[All Fields] OR "inducibility"[All Fields] OR "inducible"[All Fields] OR "Inducing"[All Fields]) AND "factor ifn gamma"[Title/Abstract]) OR "IL18"[Title/Abstract] | 14,909 |
| **50** | #48 OR #49 | 15,456 |
| **51** | #3 AND #13 AND #50 | 18 |

**Table S2**. NIH Scale Quality Rating

|  | **First Autor** | **1** | **2** | **3** | **4** | **5** | **6** | **7** | **8** | **9** | **10** | **11** | **12** | **13** | **14** | **Total** | **Grading** |
| --- | --- | --- | --- | --- | --- | --- | --- | --- | --- | --- | --- | --- | --- | --- | --- | --- | --- |
| **IL-2** | Mannangi, N. B. | 1 | 1 | 1 | 1 | 1 | NR | NR | NR | NR | 0 | 1 | CD | NR | 0 | 6(14) | fair |
|  | Khandpur, S. | 1 | 1 | 1 | 1 | 1 | 1 | 1 | NA | CD | 1 | 1 | 1 | NR | 1 | 11(14) | good |
|  | Takahashi, H. | 1 | NR | 1 | 1 | 1 | NR | CD | NA | NR | NA | 1 | CD | NR | NA | 5(14) | poor |
|  | Kaur, S. | 1 | 1 | 1 | 1 | 1 | NR | NR | CD | 1 | NA | 1 | CD | CD | 1 | 8(14) | fair |
|  | el Barnawi, N. Y. | 1 | 1 | 1 | 1 | 1 | 1 | 1 | CD | 1 | 1 | 1 | 0 | NR | 1 | 11(14) | good |
|  | Choe, Y. B. | 1 | 1 | CD | 1 | 1 | NA | NA | 1 | 1 | CD | 1 | 0 | NR | 1 | 8(14) | fair |
| **IL-4** | Chen, J. | 1 | 1 | 1 | 1 | 1 | 1 | NR | 1 | 1 | NR | 1 | 1 | NR | 1 | 11(14) | good |
|  | Khandpur, S. | 1 | 1 | 1 | 1 | 1 | 1 | 1 | NA | CD | 1 | 1 | 1 | NR | 1 | 11(14) | good |
|  | el Barnawi, N. Y. | 1 | 1 | 1 | 1 | 1 | 1 | 1 | CD | 1 | 1 | 1 | 0 | NR | 1 | 11(14) | good |
|  | Verghese, B. | 1 | 1 | 1 | 1 | 1 | 0 | NR | NR | 1 | NR | 1 | CD | NR | 1 | 8(14) | fair |
| **IL-12** | Takahashi, H. | 1 | NR | 1 | 1 | 1 | NR | CD | NA | NR | NA | 1 | CD | NR | NA | 5(14) | poor |
|  | Michalak-Stoma, A. | 1 | 0 | 1 | 1 | 1 | NA | NA | NA | 1 | 1 | 1 | 0 | NR | 1 | 8(14) | fair |
|  | Kyriakou, A. | 1 | 1 | 1 | 1 | 1 | 0 | NR | 0 | 1 | 0 | 1 | CD | NR | 1 | 8(14) | fair |
|  | Arican, O. | 1 | 1 | 1 | 1 | 1 | 1 | NA | 1 | 1 | 0 | 1 | CD | NR | 1 | 10(14) | good |
|  | Brito-Luna, M. J. | 1 | 1 | 1 | 1 | 1 | 1 | 1 | 1 | 1 | 0 | 1 | 0 | NR | 1 | 11(14) | good |
| **IL-17** | Elbana, A. M. | 1 | 1 | 1 | 1 | 1 | NA | NA | NA | NA | NA | 1 | CD | NR | 1 | 7(14) | fair |
|  | Xuan, M. L. | 1 | 1 | 1 | 1 | 1 | 0 | NR | NR | NR | NA | 1 | CD | NR | 1 | 7(14) | fair |
|  | Takahashi, H. | 1 | NR | 1 | 1 | 1 | NR | CD | NA | NR | NA | 1 | CD | NR | 1 | 5(14) | poor |
|  | Michalak-Stoma, A. | 1 | 0 | 1 | 1 | 1 | NA | NA | NA | 1 | 1 | 1 | 0 | NR | 1 | 8(14) | fair |
|  | Kyriakou, A. | 1 | 1 | 1 | 1 | 1 | 0 | NR | 0 | 1 | 0 | 1 | CD | NR | 1 | 8(14) | fair |
|  | Fotiadou, C. | 1 | 1 | 1 | 1 | 1 | 0 | NR | NR | NR | NA | 1 | CD | NR | 1 | 7(14) | fair |
|  | Choe, Y. B. | 1 | 1 | CD | 1 | 1 | NA | NA | 1 | 1 | CD | 1 | 0 | NR | 1 | 8(14) | fair |
|  | Chhabra, S. | 1 | 1 | 1 | 1 | 1 | 0 | NA | NA | NA | NA | 1 | CD | NR | 1 | 7(14) | fair |
|  | Akşan, B. | 1 | 1 | 1 | 1 | 1 | 1 | 1 | 1 | 1 | 0 | 1 | 0 | NR | 1 | 11(14) | good |
|  | Arican, O. | 1 | 1 | 1 | 1 | 1 | 1 | NA | 1 | 1 | 0 | 1 | CD | NR | 1 | 10(14) | fair |
|  | Nassar, A. A. | 1 | 1 | 1 | 1 | 1 | CD | NA | NR | NR | NR | 1 | CD | NR | 1 | 7(14) | fair |
| **IL-18** | Takahashi, H. | 1 | NR | 1 | 1 | 1 | NR | CD | NA | NR | NA | 1 | CD | NR | 1 | 5(14) | poor |
|  | Pietrzak, D. | 1 | 1 | 1 | 1 | 1 | 1 | 1 | 1 | 1 | NR | 1 | CD | NR | 1 | 11(14) | good |
|  | Pietrzak, A. | 1 | 1 | 1 | 1 | 1 | 1 | NA | NA | NA | NA | 1 | CD | NR | 1 | 8(14) | fair |
|  | Gangemi, S. | 1 | 0 | 1 | 1 | 1 | NA | NA | NA | NA | NA | 1 | CD | NR | 1 | 6(14) | fair |
|  | Arican, O. | 1 | 1 | 1 | 1 | 1 | 1 | NA | 1 | 1 | 0 | 1 | CD | NR | 1 | 10(14) | fair |
| **IL-22** | Michalak-Stoma, A. | 1 | 0 | 1 | 1 | 1 | NA | NA | NA | 1 | 1 | 1 | 0 | NR | 1 | 8(14) | fair |
|  | Fotiadou, C. | 1 | 1 | 1 | 1 | 1 | 0 | NR | NR | NR | NA | 1 | CD | NR | 1 | 7(14) | fair |
|  | Sobhan, M. R. | 1 | 1 | 1 | 1 | 1 | NR | NA | NA | NA | NA | 1 | CD | NR | 1 | 7(14) | fair |
|  | Hofny, E. R. M. | 1 | 1 | 1 | 1 | 1 | NR | NR | 1 | 1 | 0 | 1 | CD | NR | 1 | 9(14) | fair |
|  | Brito-Luna, M. J. | 1 | 1 | 1 | 1 | 1 | 1 | 1 | 1 | 1 | 0 | 1 | 0 | NR | 1 | 11(14) | good |
| **IL-23** | Michalak-Stoma, A. | 1 | 0 | 1 | 1 | 1 | NA | NA | NA | 1 | 1 | 1 | 0 | NR | 1 | 8(14) | fair |
|  | Kyriakou, A. | 1 | 1 | 1 | 1 | 1 | 0 | NR | 0 | 1 | 0 | 1 | CD | NR | 1 | 8(14) | fair |
|  | Fotiadou, C. | 1 | 1 | 1 | 1 | 1 | 0 | NR | NR | NR | NA | 1 | CD | NR | 1 | 7(14) | fair |
|  | Filiz, B. | 1 | 1 | 1 | 1 | 1 | 1 | 1 | 0 | 1 | 0 | 1 | 1 | NR | 1 | 11(14) | good |
|  | Chhabra, S. | 1 | 1 | 1 | 1 | 1 | 0 | NA | NA | NA | NA | 1 | CD | NR | 1 | 7(14) | fair |
|  | Brito-Luna, M. J. | 1 | 1 | 1 | 1 | 1 | 1 | 1 | 1 | 1 | 0 | 1 | 0 | NR | 1 | 11(14) | good |
| **IL-35** | Elbana, A. M. | 1 | 1 | 1 | 1 | 1 | NA | NA | NA | NA | NA | 1 | CD | NR | 1 | 7(14) | fair |
|  | Chen, J. | 1 | 1 | 1 | 1 | 1 | 1 | NR | 1 | 1 | NR | 1 | 1 | NR | 1 | 11(14) | good |
| **IL-36** | Sehat, M. | 1 | 1 | 1 | 1 | 1 | NA | NA | NA | NA | NA | 1 | CD | NR | 1 | 7(14) | fair |
|  | Chen, J. | 1 | 1 | 1 | 1 | 1 | 1 | NR | 1 | 1 | NR | 1 | 1 | NR | 1 | 11(14) | good |
| **IFN-γ** | Khandpur, S. | 1 | 1 | 1 | 1 | 1 | 1 | 1 | NA | CD | 1 | 1 | 1 | NR | 1 | 11(14) | good |
|  | Mannangi, N. B. | 1 | 1 | 1 | 1 | 1 | NR | NR | NR | NR | 0 | 1 | CD | NR | 0 | 6(14) | fair |
|  | Elbana, A. M. | 1 | 1 | 1 | 1 | 1 | NA | NA | NA | NA | NA | 1 | CD | NR | 1 | 7(14) | fair |
|  | Arican, O. | 1 | 1 | 1 | 1 | 1 | 1 | NA | 1 | 1 | 0 | 1 | CD | NR | 1 | 10(14) | fair |
|  | Abdallah, M. A. | 1 | 1 | 1 | 1 | 1 | NA | NA | NA | NA | NA | 1 | CD | NR | 1 | 7(14) | fair |
|  | Mawla, M. Y. M. A. | 1 | 1 | 1 | 1 | 1 | NA | NA | NA | NA | NA | 1 | CD | NR | 1 | 7(14) | fair |
|  | el Barnawi, N. Y. | 1 | 1 | 1 | 1 | 1 | 1 | 1 | CD | 1 | 1 | 1 | 0 | NR | 1 | 11(14) | good |
| **TGF-β** | Ahmed, B. T. | 1 | 1 | 1 | 1 | 1 | NR | 1 | NR | 1 | 1 | 1 | CD | NR | 1 | 10(14) | fair |
|  | Elbana, A. M. | 1 | 1 | 1 | 1 | 1 | NA | NA | NA | NA | NA | 1 | CD | NR | 1 | 7(14) | fair |
|  | Meki, A. R. | 1 | 1 | 1 | 1 | 1 | NA | NA | NA | NA | NA | 1 | CD | NR | 1 | 7(14) | fair |
|  | Zaher, H. | 1 | 1 | 1 | 1 | 1 | 0 | NR | NR | NR | 0 | 1 | CD | NR | 1 | 7(14) | fair |
| **Legend: NA (not applicable); CD (cannot determine); NR (not reported)**  **Grading for CCS: 0-4 = poor; 5-8 = fair; 9-12 = good**  **Grading for CIS: 0-5 = poor; 6-10 = fair; 11-14 = good** | | | | | | | | | | | | | | | | | |

**Supplementary Drawing. Funnel diagram and Egger diagram of circulating inflammatory cytokines.**


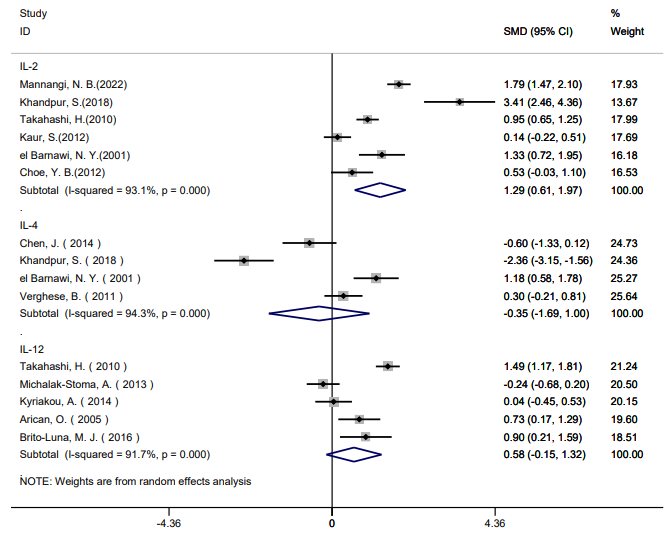


**Fig S1.** Forest plot of circulating inflammatory factors IL-2, IL-4, IL-12 and psoriasis risk (given as standard mean difference between psoriasis group and control group)


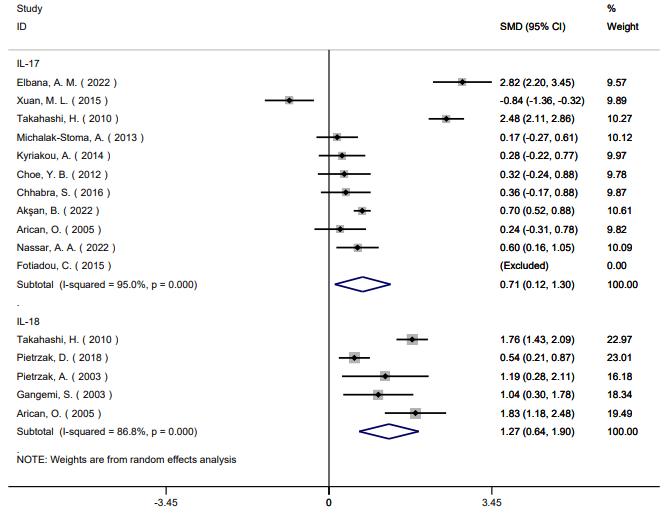


**Fig S2.** Forest plot of circulating inflammatory factors IL-17, IL-18 and psoriasis risk (given as standard mean difference between psoriasis group and control group)


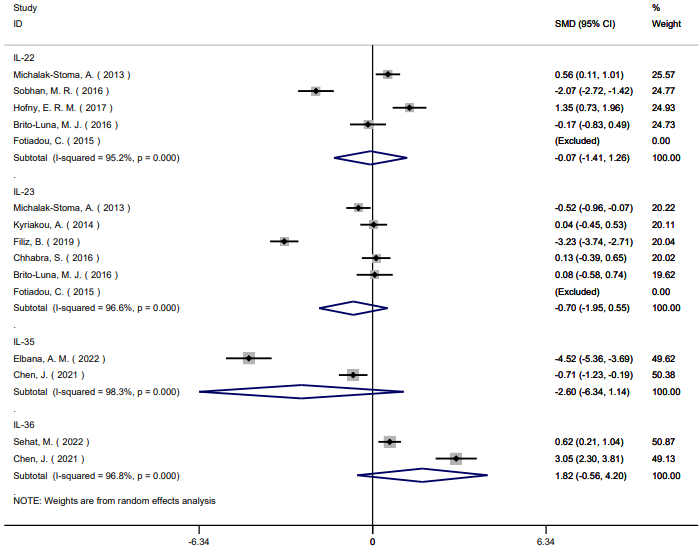


**Fig S3.** Forest plot of circulating inflammatory factors IL-22, IL-23, IL-35, IL-36 and psoriasis risk (given as standard mean difference between psoriasis group and control group)


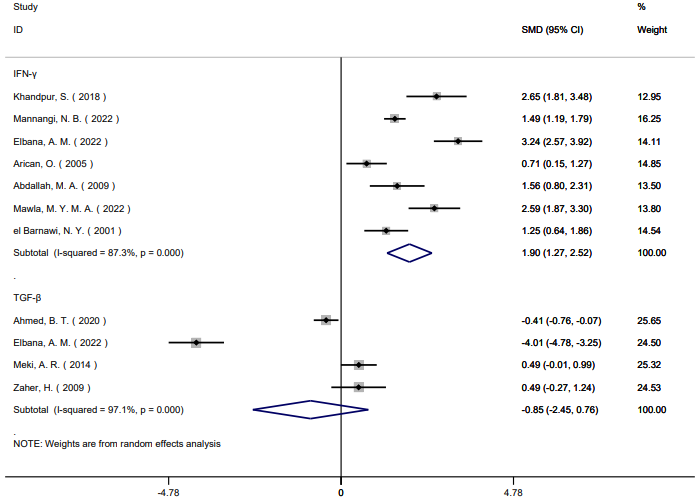


**Fig S4.** Forest plot of circulating inflammatory factors IFN-γ, TGF-β and psoriasis risk (given as standard mean difference between psoriasis group and control group)


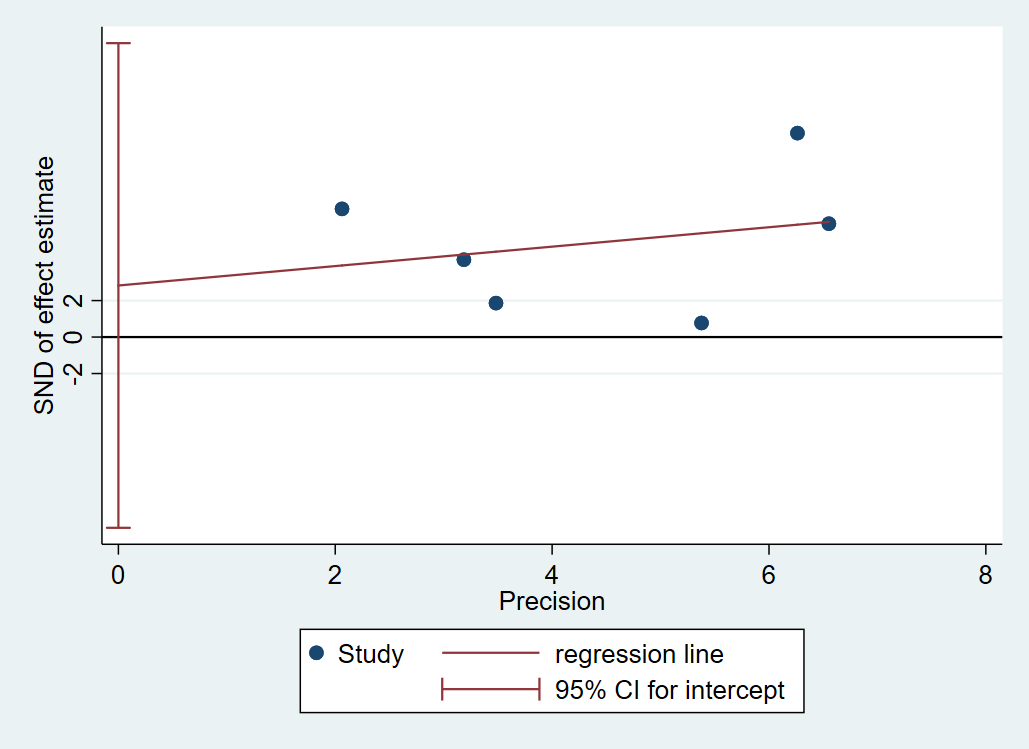


**Figure S5.** IL-2 Egger diagram


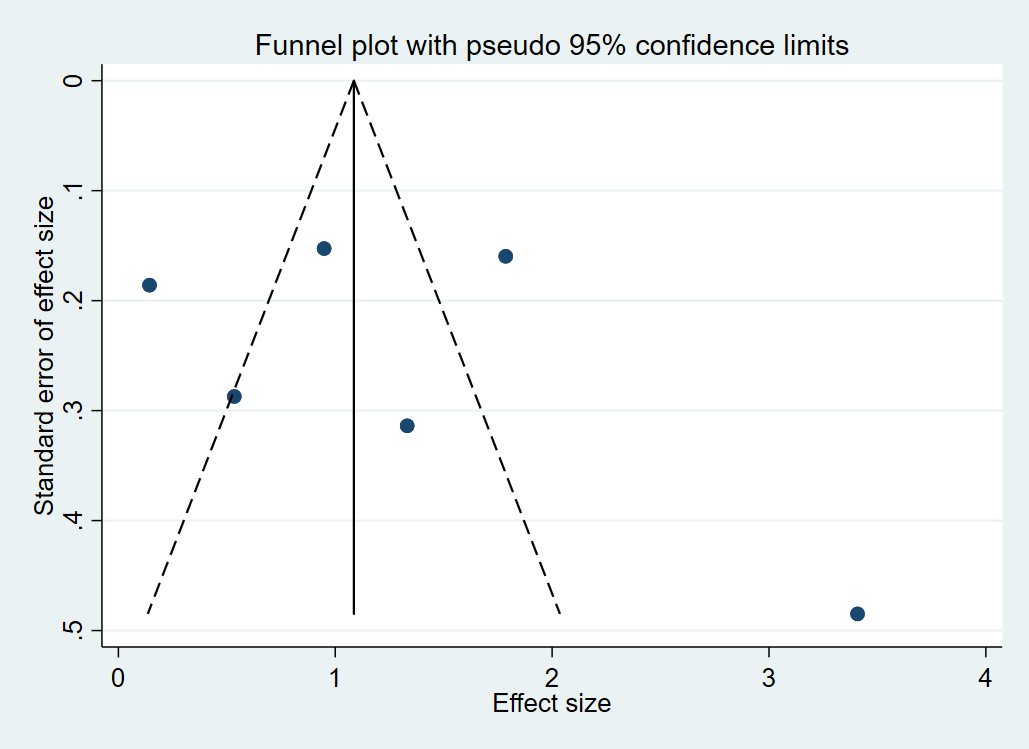


**Figure S6.** IL-2 Funnel diagram


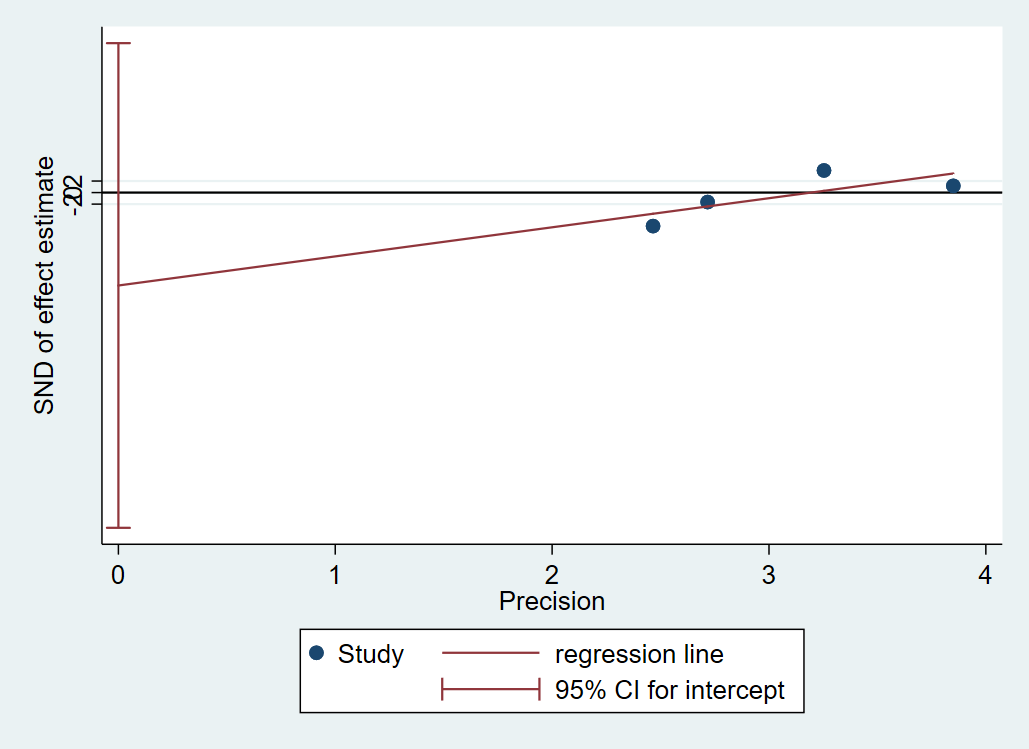


**Figure S7.** IL-4 Egger diagram


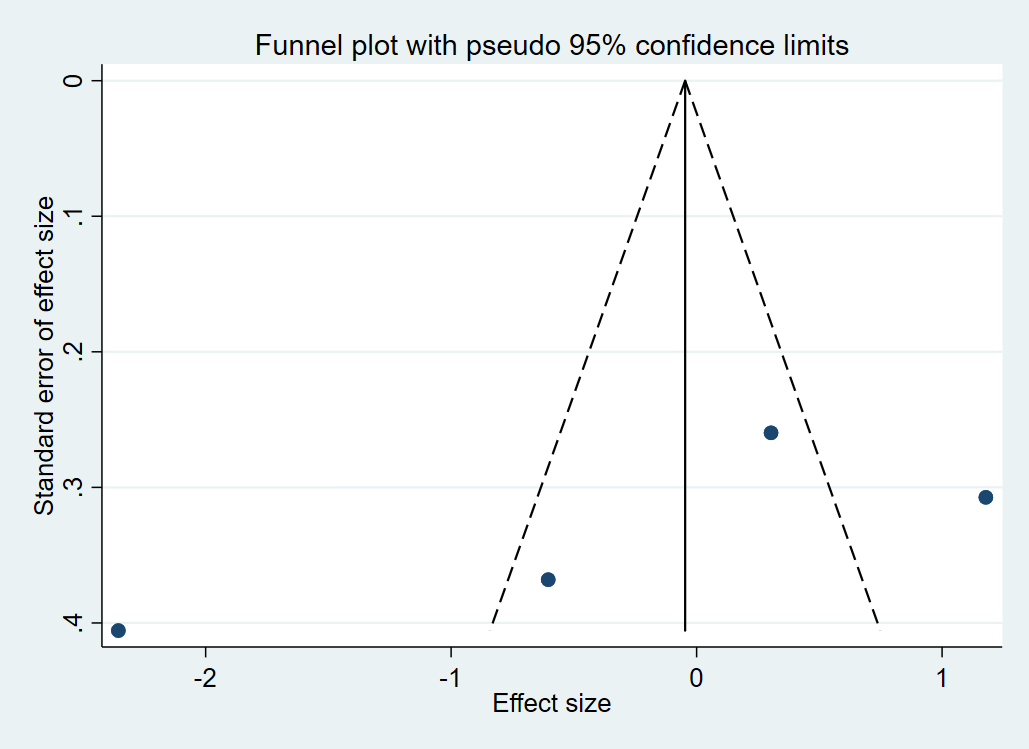


**Figure S8.** IL-4 Funnel diagram


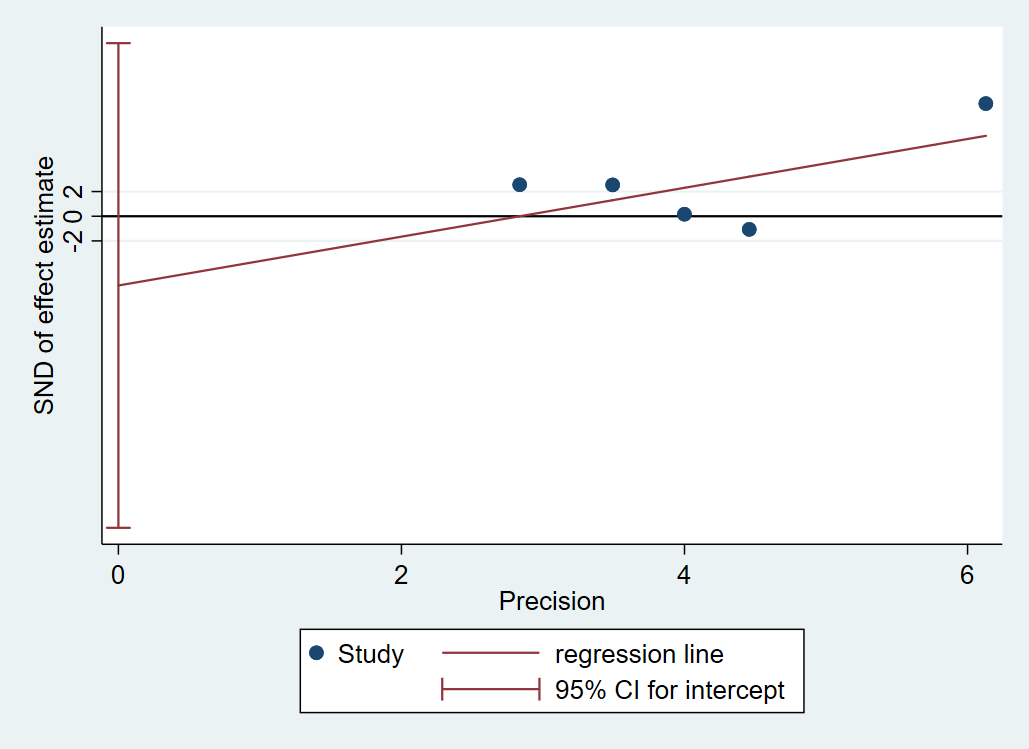


**Figure S9.** IL-12 Egger diagram


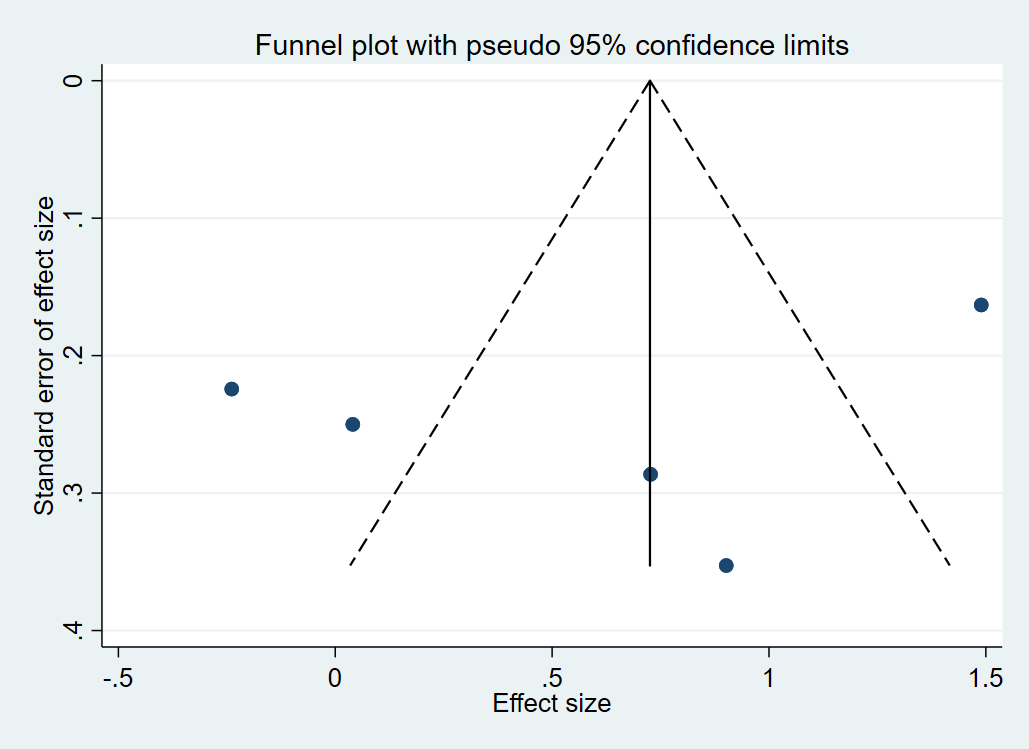


**Figure S10.** IL-12 Funnel diagram


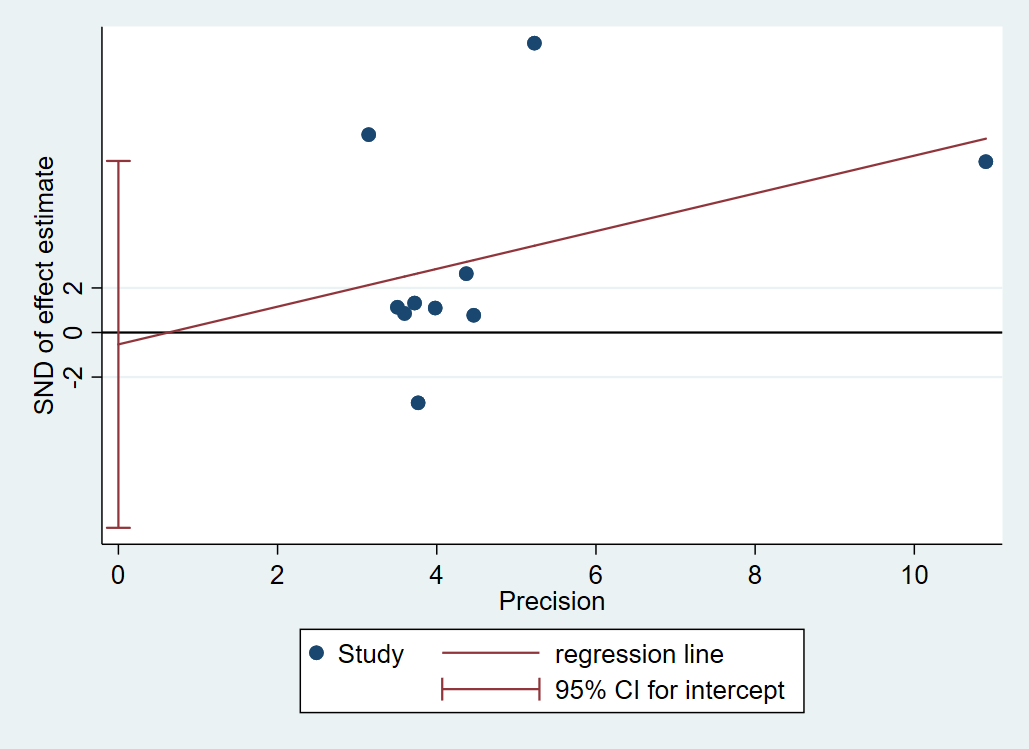


**Figure S11.** IL-17 Egger diagram


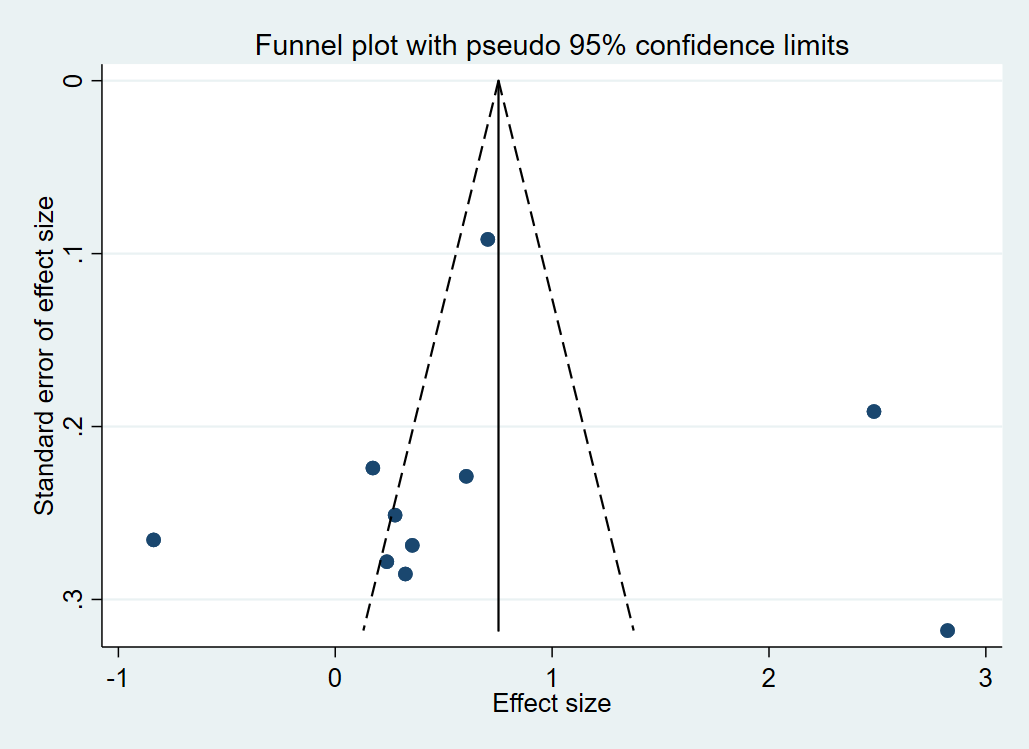


**Figure S12.** IL-17 Funnel diagram


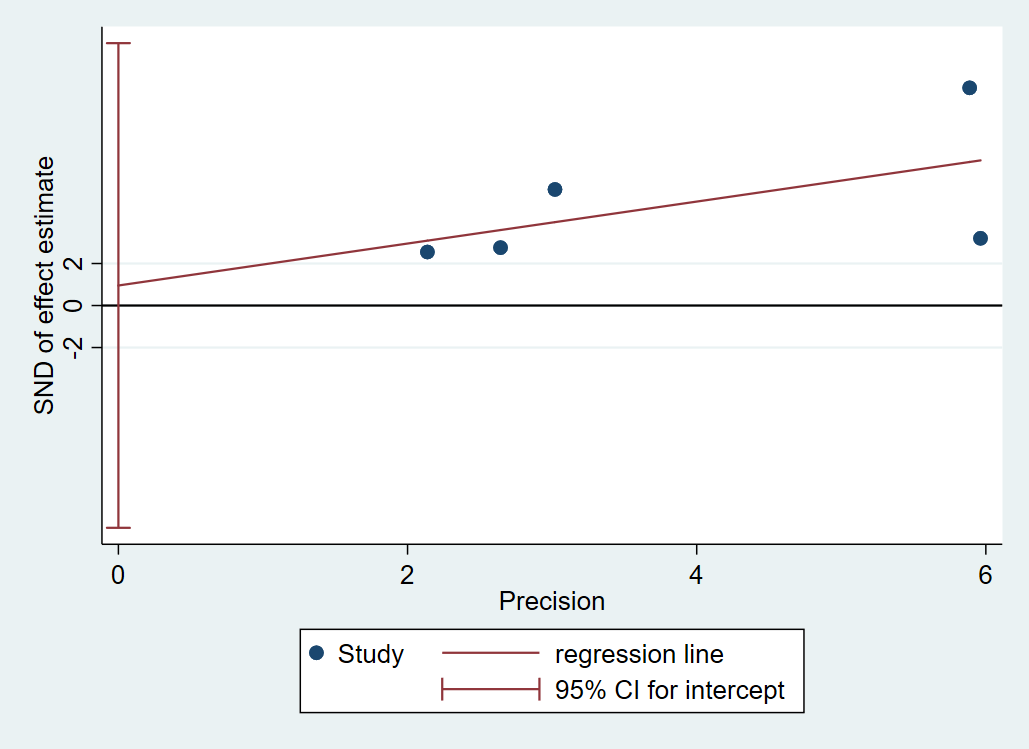


**Figure S13.** IL-18 Egger diagram


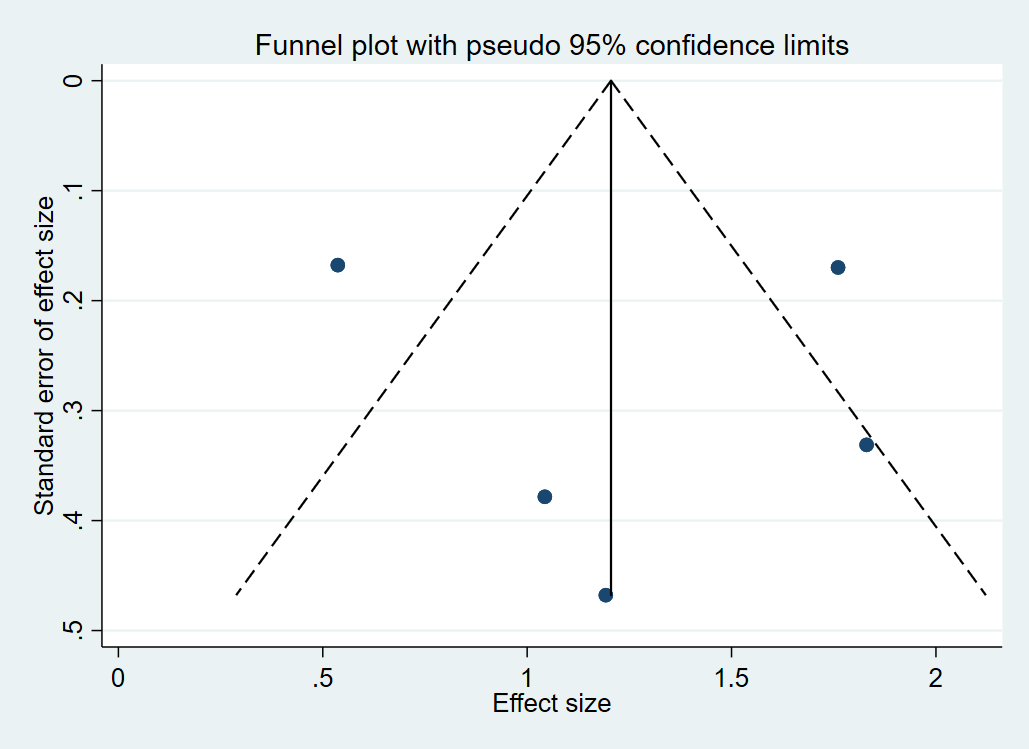


**Figure S14.** IL-18 Funnel diagram


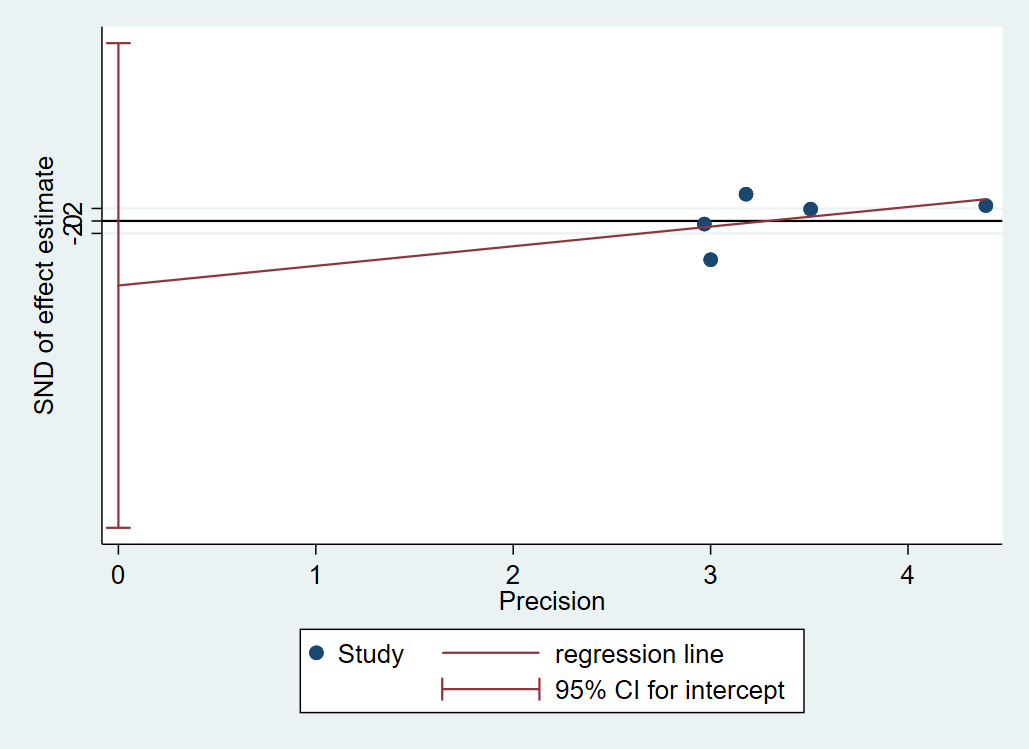


**Figure S15.** IL-22 Egger diagram


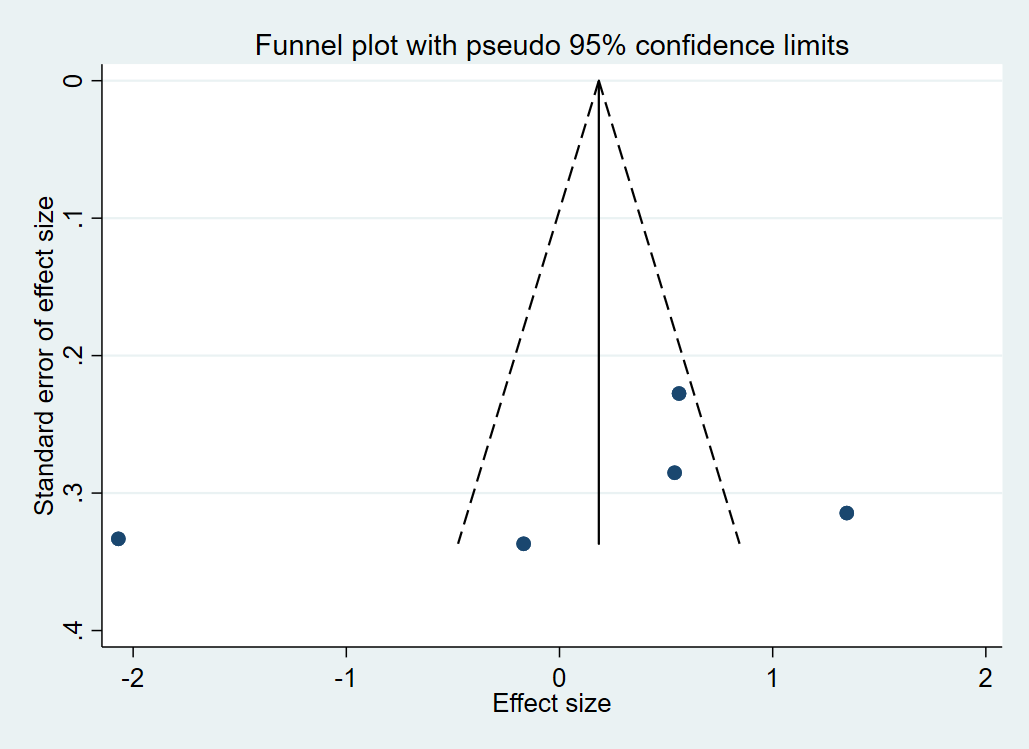


**Figure S16.** IL-22 Funnel diagram


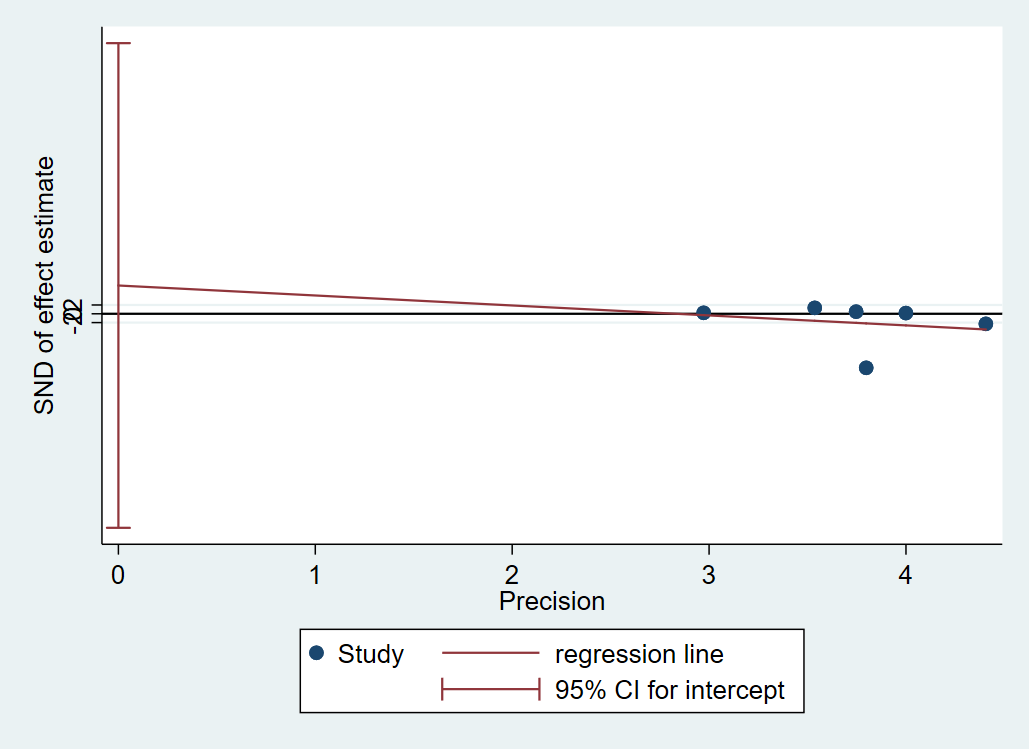


**Figure S17.** IL-23 Egger diagram


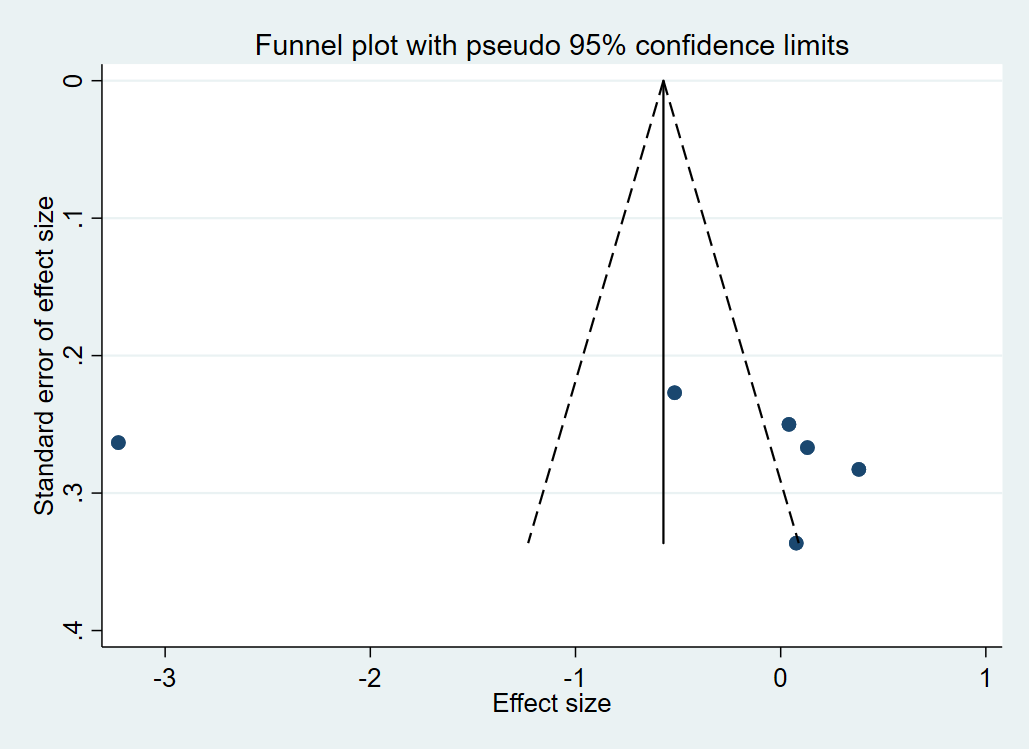


**Figure S18.** IL-23 Funnel diagram


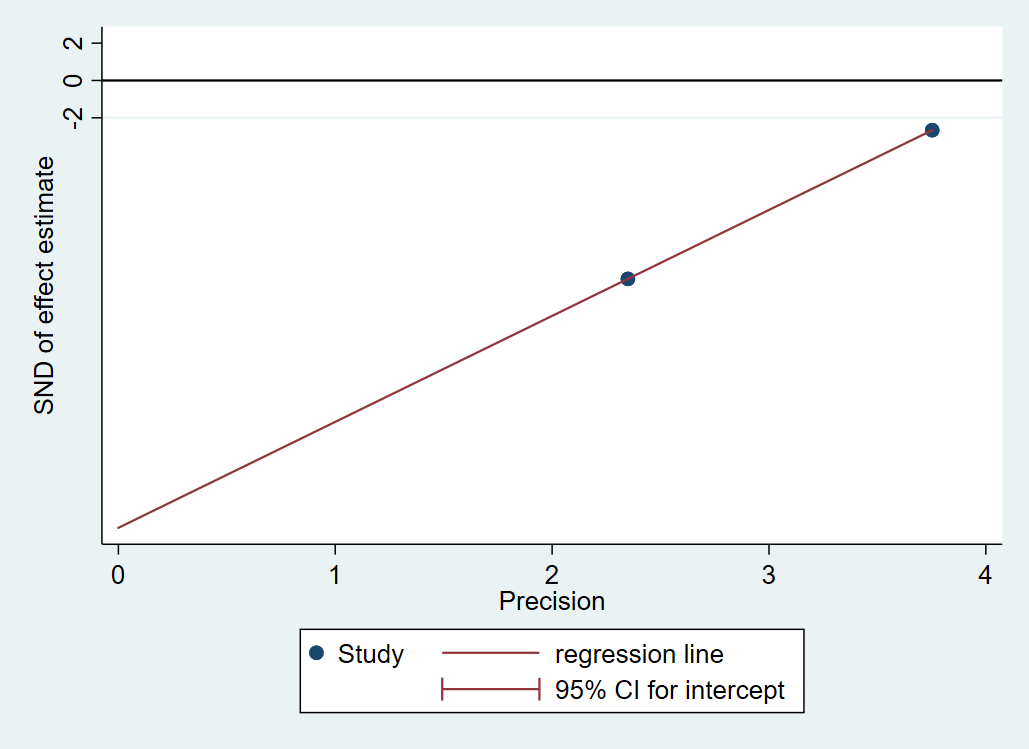


**Figure S19.** IL-35 Egger diagram


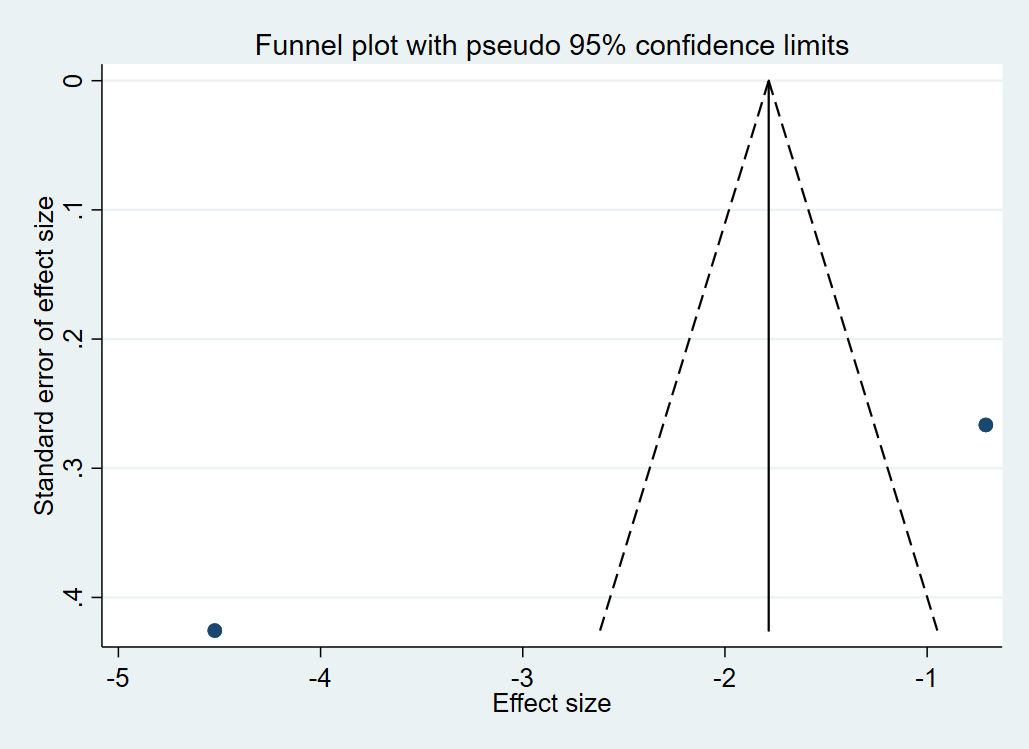


**Figure S20.** IL-35 Funnel diagram


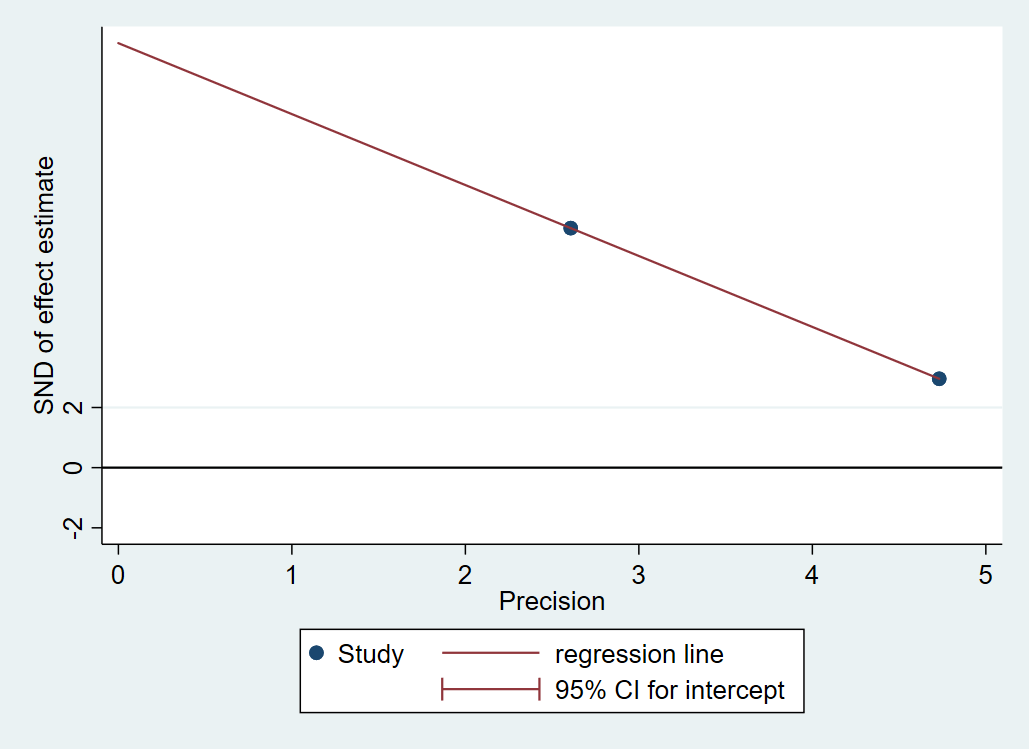


**Figure S21.** IL-36 Egger diagram


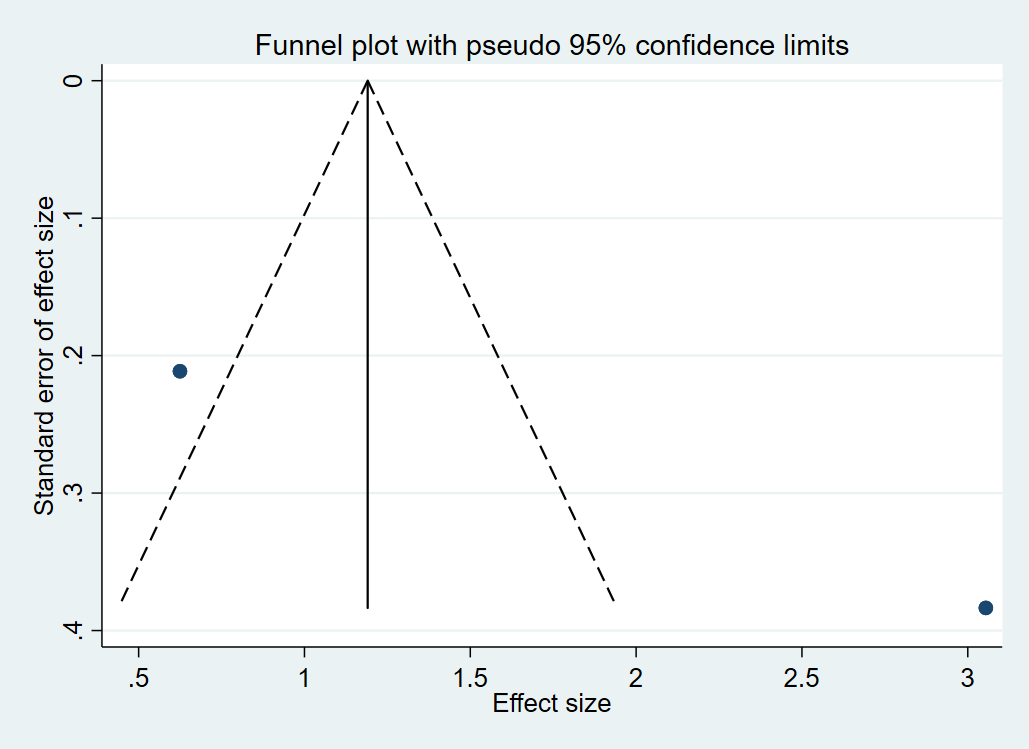


**Figure S22.** IL-36 Funnel diagram


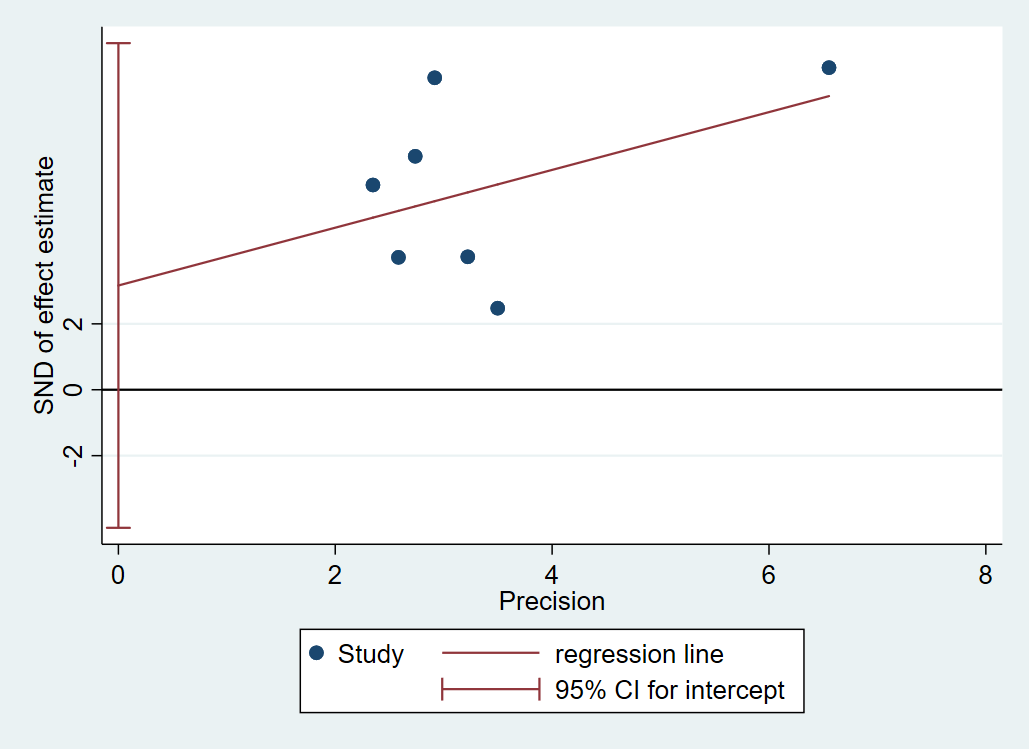


**Figure S23.** IFN-γ Egger diagram


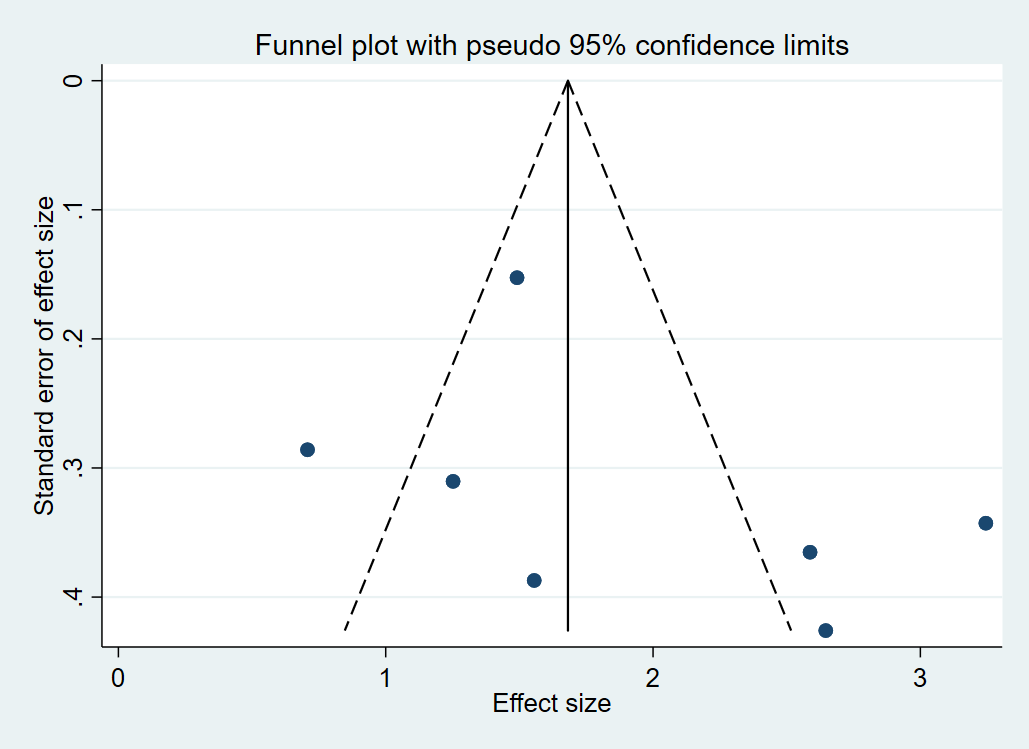


**Figure S24.** IFN-γ Funnel diagram


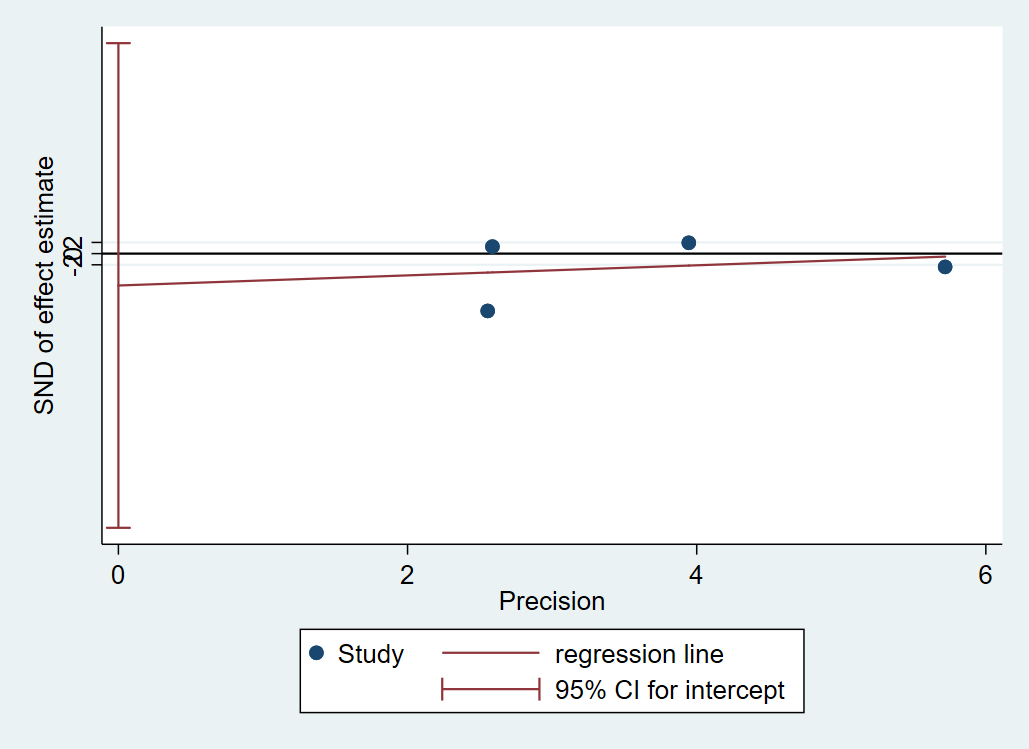


**Figure S25.** TGF-β Egger diagram


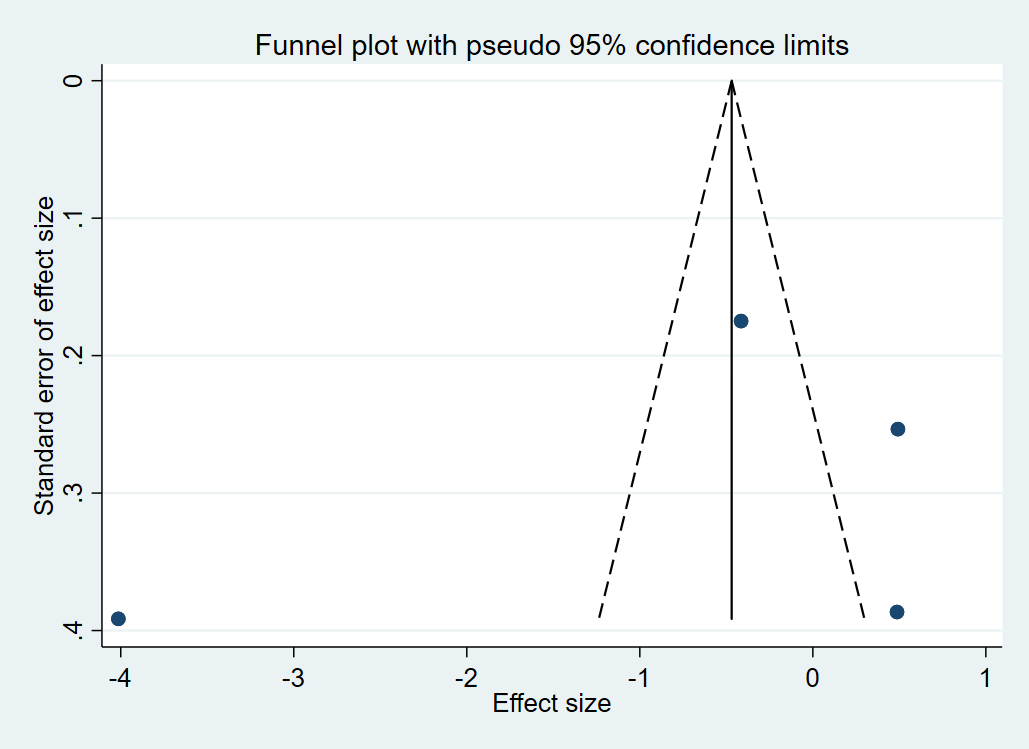


**Figure S26.** TGF-β Funnel diagram
